# Multifaceted role of *POU5F1P1* in regulating its parental stem cell gene, *POU5F1*

**DOI:** 10.1016/j.isci.2026.115137

**Published:** 2026-02-27

**Authors:** Kyohei Irie, Mitsuko Kosaka, Nobuhiko Mizuno, Ryo Omae, Yoshimasa Nakatani, Sandi Myat Noe Oo, Hisashi Masuyama, Ayano Kawaguchi

**Affiliations:** 1 Department of Human Morphology, Okayama University Graduate School of Medicine, Dentistry and Pharmaceutical Sciences, Okayama, Japan; 2 Department of Obstetrics and Gynecology, Okayama University Graduate School of Medicine, Dentistry and Pharmaceutical Sciences, Okayama, Japan

**Keywords:** Biological sciences

## Abstract

The human-specific retrogene *POU5F1P1* (OCT4-Pseudogene1; OCT4-PG1), derived from stem cell factor *POU5F1* (OCT4A), is predicted to encode an OCT4A-like protein; however, its function remains unclear. This study investigated OCT4-PG1 expression, translational control, and its role in endometrial cancer and stem cell regulation. Quantitative analyses revealed that elevated OCT4A, but not OCT4-PG1, expression correlated with clinical risk factors associated with poor prognosis in patients with endometrial cancer. OCT4-PG1 is under strong translational suppression mediated by its untranslated region and does not function as a protein under normal conditions. Instead, it acts as a non-coding RNA that suppresses OCT4A translation. Structural analyses showed that a single amino acid deletion (Gln259) destabilizes the OCT4-PG1 protein, thereby preventing its tumorigenic and transcriptional functions. Nevertheless, OCT4-PG1 forms heterodimers with OCT4A or SOX2, enhancing the regulatory activity of OCT4A. These findings highlight the regulatory role of pseudogenes in cancer and stem cell biology, with implications for therapies targeting OCT4A-related pathways.

## Highlights

• OCT4-PG1 expression inversely correlates with endometrial cancer grade
• OCT4-PG1 5′UTR strongly inhibits OCT4A translation
• Single amino acid deletion destabilizes OCT4-PG1 protein
• OCT4-PG1 protein enhances OCT4A function via heterodimerization

## Graphical abstract

**Figure:**
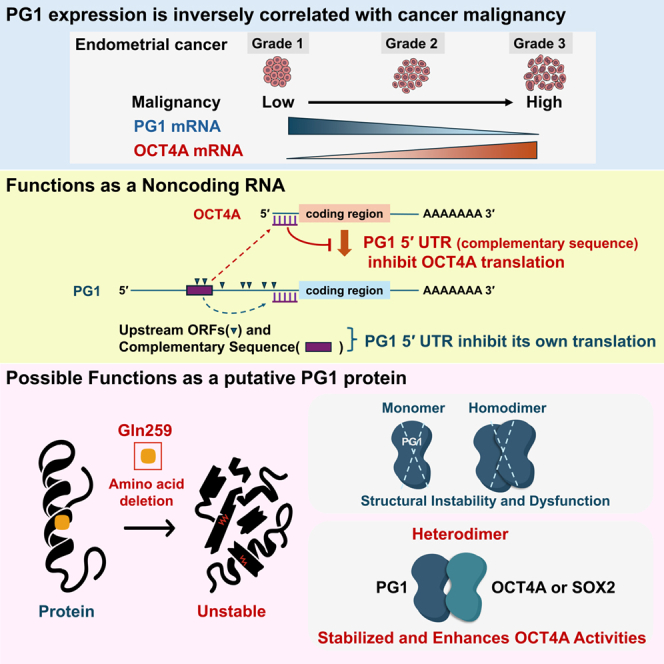

## Introduction

Pseudogenes, once considered “junk DNA” with no functional significance, are now recognized as evolutionary byproducts with potential roles in various biological processes, including cancer.^1^^,^^2^^,^^3^ Among the approximately 14,000 pseudogenes identified in the human genome, an estimated 20% may have translational potential.^4^ However, the biological functions of pseudogene-derived proteins remain largely unknown owing to limited research and technical challenges in distinguishing pseudogenes from their highly homologous parental genes with high accuracy.^1^^,^^3^

*OCT4A*, a key transcription factor essential for maintaining pluripotency, plays a critical role in early embryonic development and the maintenance of the mammalian germline.^5^^,^^6^^,^^7^^,^^8^ Furthermore, it serves as a recognized biomarker for germ cell tumors and a potential indicator of cancer stem cells (CSCs).^9^^,^^10^^,^^11^^,^^12^^,^^13^ However, the presence of multiple transcript variants of *OCT4* and its highly homologous pseudogenes complicates its detection and precise characterization.^14^^,^^15^^,^^16^^,^^17^ Additionally, the expression pattern of *OCT4* transcripts differs between human and murine somatic cells, rendering murine models unsuitable for elucidating the role of *OCT4A* in human somatic tissues and CSCs.^18^^,^^19^

The human genome contains at least eight *OCT4* pseudogenes, several of which are highly transcribed in various cancers.^14^^,^^16^^,^^20^ Among these, *POU5F1P1* (*POU5F1B, OCT4-PG1*, hereafter referred to as *PG1*), located in the frequently amplified 8q24 region, shares the highest sequence similarity with OCT4A and is predicted to encode a protein with a similar structure. Elevated PG1 expression has been associated with poor prognosis in several cancers, including gastric and colorectal cancers.^21^^,^^22^^,^^23^ Given its high open reading frame (ORF) homology with *OCT4A, PG1* is hypothesized to perform similar functions in CSCs. However, previous studies have indicated that PG1 exhibits little to no transcriptional regulatory activity in the nucleus.^21^^,^^24^ Recent findings suggest that *PG1* is localized in the cytoplasm rather than in the nucleus and may contribute to colorectal cancer progression, indicating a distinct and unexpected function compared to that of *OCT4A*.^22^ These contradictory findings raise fundamental questions regarding whether *PG1* is functionally active, and if so, what is its mechanism of action.

The high sequence homology between *OCT4A* and *PG1* has led to methodological challenges, including the use of nonspecific PCR primers, antibodies, and siRNA reagents, resulting in unreliable results. These technical limitations hinder the precise characterization of *OCT4A* and *PG1* in cancer. In a previous study, we developed a highly reliable method capable of eliminating false-positive results to detect authentic OCT4 transcripts and their translational products. This method has conclusively demonstrated the existence of an OCT4-positive subpopulation of human cancer cells and its association with enhanced migratory and invasive properties.^19^^,^^25^ However, whether *PG1* is equivalent to *OCT4A* or shares any biological roles with *OCT4A* remains unclear.

To clarify the function of PG1 and its relationship with *OCT4A*, this study aimed to determine whether PG1 acts as a coding or non-coding RNA, assess its correlation with endometrial cancer malignancy, and investigate how specific amino acid residues influence its structural and functional properties. By resolving inconsistencies in prior research and applying a highly reliable detection methodology, we sought to provide novel insights into the biological role of PG1 and its potential implications in cancer and stem cell biology.

## Results

### Public databases indicate distinct expression patterns of OCT4 and PG1 in cancer cells and tumor tissues

**Figure 1:**
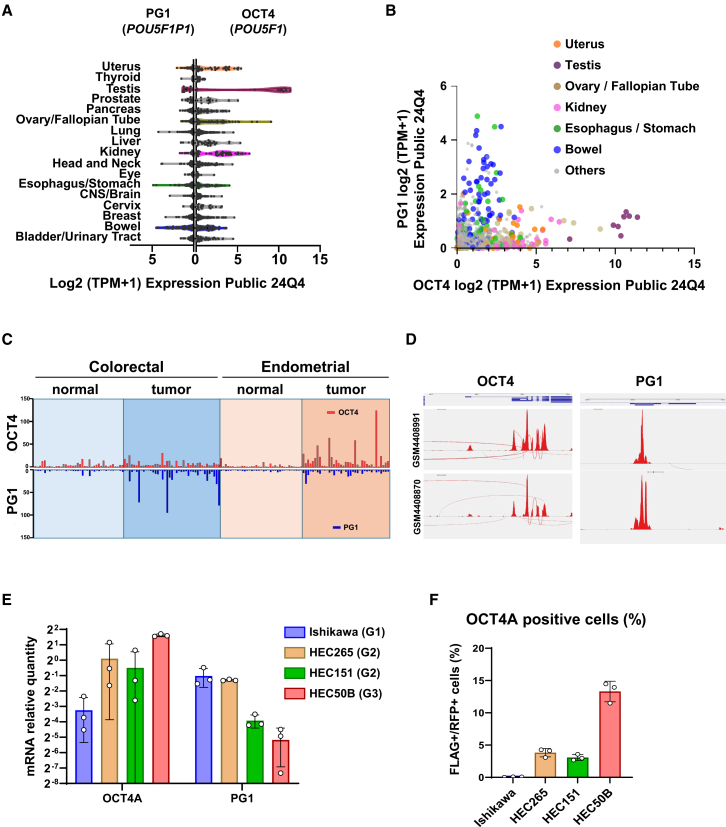
Differential expression of OCT4A and PG1 in uterine cancer cell lines (A) OCT4 and PG1 gene expression data from cancer cell lines were registered in the Public 24Q4 data of the Cancer Cell Line Encyclopedia (CCLE). The Figure plots the data from representative lineage cell lines. (B) Scatterplot of the correlation between OCT4A and PG1 expression. Pearson’s correlation coefficient between OCT4A and PG1 across all cell lines was 0.207. The correlation within the bowel lineage alone was 0.359, and within the uterine lineage alone, it was 0.126. In the combined group of the testes, ovary/fallopian tube, kidney, uterus, esophagus/stomach, and bowel, the correlation was 0.06364, indicating a lack of correlation between the two parameters. (C) Evaluation of OCT4 and PG1 gene expression levels in normal and cancerous tissue samples from patients with uterine and colorectal cancers using Gene Expression Omnibus 2R (GEO2R) against the GSE146889 RNA-sequencing database. (D) Sashimi plots of samples with high OCT4 and PG1 expression in GSE146889. Sashimi plots were used to visualize splice junctions derived from the aligned RNA-seq data along a gene annotation track. In these plots, the coverage of each alignment track is represented by a bar graph. Arcs connect exons to indicate splice junctions, and the number of reads spanning each junction is displayed on the arcs. (E) The relative mRNA levels of OCT4A and PG1 were measured using real-time PCR with specific primers (Figures S1D and S1E). Data are presented as relative expression levels normalized to HPRT1, with HPRT1 set to 1. *n* = 3. Mean ± SD. Data from colorectal cancer and ovarian germ cell tumor cell lines, where high OCT4A expression is known, are shown in Figure S2. (F) Validation of OCT4A-positive endometrial cancer cell lines using tagged genomic transgenes. Immunocytochemistry was performed using an anti-FLAG antibody on cells that were transfected with pOCT4AGen-FLAG. Anti-FLAG antibodies and RFP markers were used to identify FLAG-tagged OCT4A protein-positive and vector-transfected cells, respectively (Figure S3). Proportion of FLAG/RFP-positive cells in each cell line. The percentages of FLAG-positive cells relative to RFP-positive cells were 0.05, 3.83, 3.08, and 13.3% in Ishikawa, HEC265, HEC151, and HEC50B cells, respectively. *n* = 3. Mean ± SD.

Using the Cancer Cell Line Encyclopedia (CCLE) database (DepMap 24Q4 Public^26^), we examined the expression levels of OCT4 and PG1 transcripts in various human cancer cell lines derived from different tissues (Figures 1A, 1B, S1A, and S1B). In addition to reproductive system cancers (testis and ovary/fallopian tube), OCT4 was highly expressed in uterine and kidney cancers, whereas PG1 was highly expressed in esophageal, stomach, and bowel cancers (Figure 1A). Gene expression analysis revealed a weak correlation between OCT4 and PG1 expression in bowel cancer cell lines (r = 0.36) and an even lower correlation in uterine cancer cell lines (r = 0.13). No significant correlation was observed between OCT4 and PG1 expression in the ovary/fallopian tube, kidney, uterus, esophagus, stomach, and bowel (r = 0.064) (Figure 1B).

To further investigate OCT4 and PG1 expression in cancer tissues, we analyzed the GSE146889 dataset using GEO2R.^27^^,^^28^ Both genes were expressed at higher levels in cancer tissues than in normal tissues. Analysis of cancer tissue datasets revealed high OCT4 expression in endometrial cancer and predominant PG1 expression in colorectal cancer (Figure 1C). However, these results were inconclusive because both OCT4 and PG1 have multiple transcript variants (Figure S1C). The OCT4A isoform functions as a transcriptional regulator that is essential for maintaining pluripotency. To gain further insight, we analyzed the splicing patterns of OCT4A transcripts and found that exon 1–2 splicing, which is specific to OCT4A, was barely detected (Figure 1D), indicating the need for a more direct quantification. Therefore, we conducted a quantitative analysis to measure the expression levels of OCT4A and PG1 in endometrial cancer.

### OCT4A expression correlates with malignancy, whereas PG1 expression is inversely associated with endometrial cancer cell lines

To assess the relationship between OCT4A and PG1 expression and malignancy, we quantitatively analyzed their transcript levels in four endometrial cancer cell lines representing different grades: Ishikawa (grade 1 [G1]), HEC265 (grade 2 [G2]), HEC151 (grade 2 [G2]), and HEC50B (grade 3 [G3]).^29^^,^^30^ Real-time PCR was performed using primers specifically designed to distinguish OCT4A and PG1 with high accuracy (Figures 1D and S1D–S1E). We observed a trend in which OCT4A expression increased with malignancy grade, whereas PG1 expression decreased (Figure 1E). Compared to PA1, a human ovarian teratocarcinoma-derived embryonic carcinoma cell line known for its high OCT4A expression and used to analyze cancer stem cell characteristics, the expression level of OCT4A in endometrial cancer cell lines was lower. Compared to colorectal cancer cell lines, which exhibited high PG1 expression, endometrial cancer cell lines showed lower PG1 levels (Figure S2).

To further investigate the relationship between OCT4A expression and malignancy, we used a previously reported genomic transgene method^19^ to quantify the proportion of OCT4A-positive cells. A strong correlation was observed between the positivity rate and OCT4A expression levels in endometrial cancer cell lines (Figures 1F and S3). PG1 expression, in contrast, showed an inverse relationship with malignancy.

### OCT4A and PG1 expression patterns in early-stage endometrial cancer tissues

To further investigate the clinical relevance of OCT4A and PG1 expression, we analyzed their transcript levels in clinical specimens (*n* = 61) of FIGO stage I endometrial cancer (Table S1).

**Figure 2:**
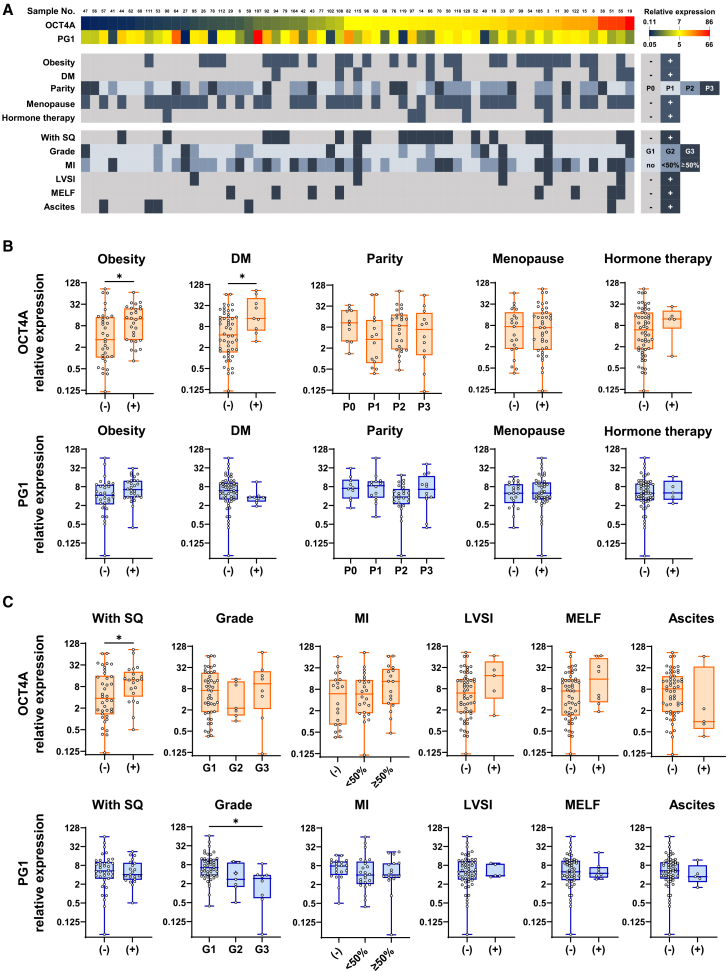
Quantitative analysis of OCT4A and PG1 expression in early-stage endometrial cancer tissue samples (A) The gene expression levels of OCT4A and PG1 in each clinical sample and their respective clinical and pathological background findings are shown. Patient background information is presented in Table S1. Gene expression levels are represented as 2^−ΔCT^ values using HPRT1 as the endogenous control. The color intensity in the Figure represents the relative gene expression levels. The expression level data for each sample are shown in Figure S4. (B) OCT4A and PG1 expression and their correlation with the clinical background. (C) OCT4A and PG1 expression and their correlations with pathological findings. Each data point represents the mean of the triplicate measurements. Statistical processing was performed using the Mann-Whitney U and Kruskal-Wallis tests. Statistical significance was defined as *p* < 0.05. ∗ = *p* < 0.05, ∗∗ = *p* < 0.01, ∗∗∗ = *p* < 0.001, and ∗∗∗∗ = *p* < 0.0001. (−) = absent, (+) = positive. DM, diabetes mellitus; SQ, squamous differentiation; MI, myometrial invasion; CI, cervical stromal invasion; LVSI, lymphovascular space invasion; MELF, microcystic, elongated, and fragmented; ascites, ascites cytology.

The expression levels, patient background, and pathological findings are shown in Figures 2A and S4. We examined the patients’ background characteristics, including obesity, menopause, diabetes mellitus (DM), and hormone therapy, all of which are established risk factors for endometrial cancer. High OCT4A expression was significantly correlated with DM (Mann-Whitney test; *p* = 0.0191) and obesity (Mann-Whitney test; *p* = 0.0194), whereas PG1 expression showed no correlation with any background variable (Figure 2B).

A significant association was observed between elevated OCT4A expression and the presence of squamous differentiation (SQ; Mann-Whitney test; *p* = 0.0374). Although no statistically significant differences were found for G3, myometrial invasion (MI ≥ 1/2), lymphovascular space invasion (LVSI), or the microcystic, elongated, and fragmented (MELF) pattern, a trend toward higher OCT4A expression was noted (Figure 2C). In contrast, PG1 expression was inversely correlated with tumor grade (Kruskal-Wallis test; G1 vs. G3: *p* = 0.0175) (Figure 2C). No other pathological features were associated with PG1 expression (Figure 2C).

### PG1 translation is repressed by its 5′ UTR

**Figure 3:**
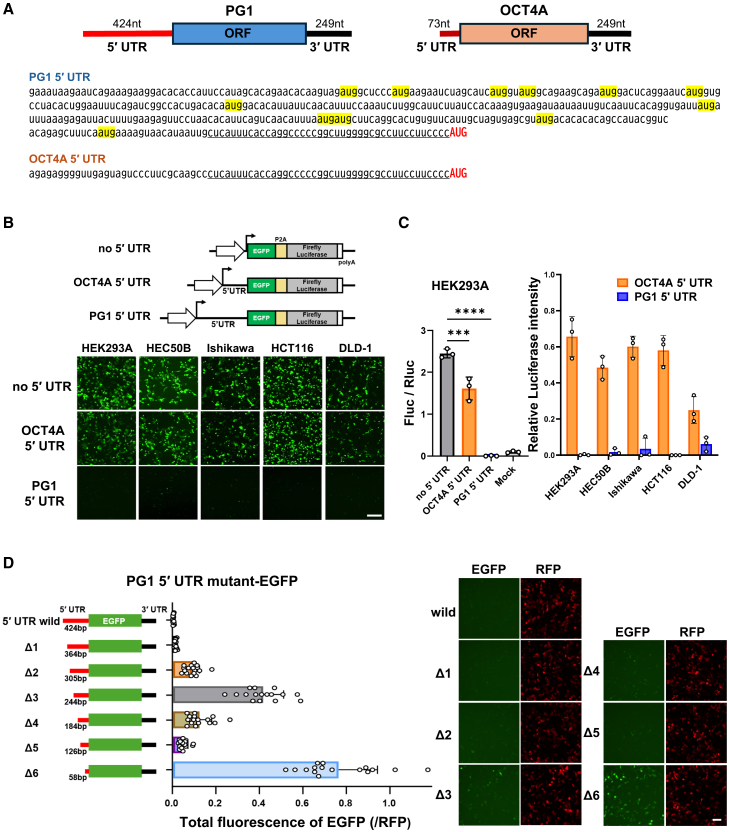
5′ UTR of PG1 mRNA suppresses its translation (A) The 5′ UTR sequences of PG1 and OCT4A were analyzed to assess their effects on protein translation. The start codon AUG is shown in upper case (red), whereas the upstream AUG codons are highlighted in yellow. The underlined region indicates the sequence shared by both. (B) Upper: Diagram of each DNA construct transfected into cells. Lower: Images of EGFP fluorescence in human embryonic kidney (HEK293A), endometrial cancer (HEC50B, Ishikawa), and colorectal cancer (HCT116, DLD-1) cell lines 2 days after transfection. Scale bars, 50 μm. (C) Luminescence intensity was measured using a dual-luciferase assay. Left: Firefly/Renilla luciferase ratio in HEK293A cells. Right: Relative luminescence values normalized to the no 5′ UTR data (set to 1) for each cell line. *n* = 3. Mean ± SD. One-way ANOVA. ∗ = *p* < 0.05, ∗∗ = *p* < 0.01, ∗∗∗ = *p* < 0.001, and ∗∗∗∗ = *p* < 0.0001. (D) Fluorescence intensity values were normalized to RFP fluorescence when plasmid DNA containing EGFP and 3′ UTR sequences with different lengths of the PG1 5′ UTR sequence was transfected into HEK293A cells. The mean and variability of the 16 randomly captured fields are shown. *n* = 16. Mean ± SD. This experiment was repeated multiple times to confirm that similar results were obtained. Fluorescence images were captured 2 days after transfection. Scale bars, 100 μm.

Multiple transcript variants of PG1 have been identified in previous 5′ RACE experiments.^31^ This analysis identified at least 12 PG1 variants containing multiple exons, two of which were common across all forms and located on the 3′ side (Figure S1C). Given this, we focused on the most 3′-proximal exon, which contained a 5′ UTR of 424 bp, a major ORF of 1080 bp, and a 3′ UTR of 264 bp, spanning the entire region (Figure 3A). If the 5′ UTR affects the downstream ORF, further investigation of upstream non-coding exons is not necessary. The PG1 5′ UTR (−424 to −1) includes 12 upstream open reading frames (uORFs) (Figure 3A), which are hypothesized to play a significant role in the regulation of translation efficiency.

To examine the role of the 5′ UTR in PG1 translation, we used a plasmid construct containing EGFP-P2A-Luciferase (LUC), which was used to measure translation efficiency. This plasmid was introduced into various cultured cell lines to evaluate 5′ UTR-mediated regulation in different cellular environments. Notably, cells transfected with the PG1 5′ UTR construct exhibited minimal EGFP fluorescence (Figure 3B). To quantify translation efficiency, firefly luciferase (Fluc) activity was measured using a dual-luciferase assay. In HEK293A cells, Fluc activity in the PG1 5′ UTR construct was undetectable, with an average value of 0.056 (SD = 0.023), comparable to that of the no-transfection control (0.101, SD = 0.021). This activity was markedly lower than that observed for the OCT4A 5′ UTR (1.66, SD = 0.280) and no-5′ UTR controls (2.49, SD = 0.108) (Figure 3C, left panel). Across all tested cell lines, including endometrial and colorectal cancer cells, OCT4A consistently exhibited higher Fluc activity than did PG1 (Figure 3C, right panel).

To determine the specific region within the PG1 5′ UTR responsible for translation suppression, we generated a series of deletion mutants by removing approximately 60 bp from the 5′ end (Figure 3D, Δ1–Δ6). The average EGFP fluorescence intensity in the unmodified PG1 5′ UTR construct was 0.002 (SD = 0.002) in HEK293A cells. Following sequential deletions, fluorescence intensity gradually increased: Δ1 showed an average of 0.012 (SD = 0.004), while Δ2 exhibited 0.090 (SD = 0.035), indicating that translation repression persisted up to Δ2. However, in Δ3, the fluorescence intensity considerably increased to 0.409 (SD = 0.095). This increase was followed by a decrease in Δ4 (0.104, SD = 0.056) and Δ5 (0.046, SD = 0.020), suggesting that translation suppression was re-established in these mutants. Finally, Δ6 exhibited the highest fluorescence intensity (0.697, SD = 0.181), indicating a reduced translational suppression. The positional distribution of uORFs within the PG1 5′ UTR is illustrated in Figure S5A.

### 5′ UTR of PG1 suppresses OCT4A translation

**Figure 4:**
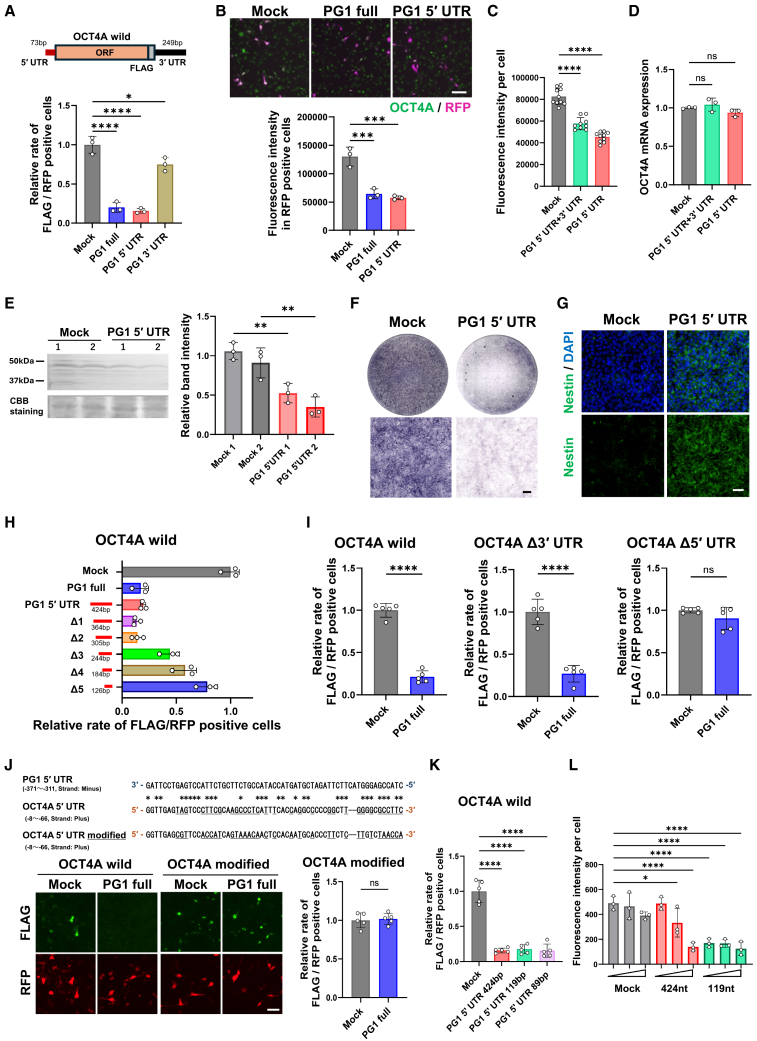
5′ UTR sequence of PG1 interacts with the 5′ UTR of OCT4A, leading to translational repression (A) Fluorescence intensity of FLAG immunostaining in NIH3T3 cells transfected with OCT4A-FLAG containing UTR, PG1 with UTR (PG1 full), or its 5′ UTR (PG1 5′ UTR) or 3′ UTR (PG1 3′ UTR) sequences. Transfection efficiency was normalized by dividing the fluorescence intensity of the co-transfected pRFP by *n* = 3. Mean ± SD. One-way ANOVA. ∗ = *p* < 0.05, ∗∗ = *p* < 0.01, ∗∗∗ = *p* < 0.001, and ∗∗∗∗ = *p* < 0.0001. (B) Immunostaining with a specific OCT4 antibody (C-10; sc5279) was performed when full-length PG1 (PG1 full) and PG1 5′ UTR sequence plasmids were transfected into PA1 cells. To verify the transfected cells, pRFP was co-transfected, and the fluorescence signal intensity of OCT4A immunostaining was quantified in RFP-positive cells. Scale bars, 100 μm. *n* = 3. Mean ± SD. One-way ANOVA. ∗ = *p* < 0.05, ∗∗ = *p* < 0.01, ∗∗∗ = *p* < 0.001, and ∗∗∗∗ = *p* < 0.0001. (C) Immunostaining with a specific OCT4A antibody (C-10; sc5279) when the RNA of the PG1 5′ UTR or 3′ UTR sequence was transfected into PA1 cells. *n* = 10 (calculated views). Mean ± SD. One-way ANOVA. ∗ = *p* < 0.05, ∗∗ = *p* < 0.01, ∗∗∗ = *p* < 0.001, and ∗∗∗∗ = *p* < 0.0001. Similar results were obtained in three independent experiments. (D) Quantity of OCT4A mRNA when the UTR sequence of PG1 was transfected into PA1 cells. Relative mRNA levels were measured using real-time PCR with specific primers (Figures S1D and S1E). Data are presented as relative expression levels normalized to HPRT1, with HPRT1 set to 1. *n* = 3. Mean ± SD. One-way ANOVA. ∗ = *p* < 0.05, ∗∗ = *p* < 0.01, ∗∗∗ = *p* < 0.001, and ∗∗∗∗ = *p* < 0.0001; ns = not significant. (E) Western blot analysis of OCT4A protein levels following the transfection of PG1 5′ UTR RNA into PA1 cells. PA1 cells were transfected with the PG1 5′ UTR sequence RNA at two concentrations (1: 5 nM and 2: 10 nM). Protein expression was detected using an OCT4A-specific antibody (C-10; sc5279). The graph on the right displays the quantification of Western blot band intensity from three independent experiments. Band intensities were normalized against the total protein amount visualized by Coomassie Brilliant Blue (CBB) staining of the gel, which served as a loading control. *n* = 3. Mean ± S.D. One-way ANOVA. ∗ = *p* < 0.05, ∗∗ = *p* < 0.01, ∗∗∗ = *p* < 0.001, and ∗∗∗∗ = *p* < 0.0001. (F and G) ALP staining images (E) and nestin immunostaining (F) after the introduction of PG1 5′ UTR RNA into PA1 cells. Scale bars, 50 μm. (H) Fluorescence intensity of FLAG immunostaining following forced expression of full-length OCT4A-FLAG and full-length PG1, mutants with the 5′ UTR shortened by 60 nt were transfected into NIH3T3 cells. *n* = 3. Mean ± SD. (I) Relative positive rate of FLAG immunostaining following forced expression of full-length PG1 and full-length OCT4A-FLAG, 5′ or 3′ UTR deleted OCT4A-FLAG, transfected into NIH3T3 cells. *n* = 5. Mean ± SD. Unpaired two-tailed Student’s *t* test. ∗ = *p* < 0.05, ∗∗ = *p* < 0.01, ∗∗∗ = *p* < 0.001, and ∗∗∗∗ = *p* < 0.0001. (J) Fluorescence intensity of FLAG immunostaining following forced expression of full-length PG1 and full-length OCT4A, and OCT4A mutants with mutations in their 5′ UTR sequence, were transfected into NIH3T3 cells. Upper: Comparison of the sequence information of the 5′ UTRs of OCT4A and PG1, where the orientation of PG1 is complementary to that of OCT4A. The common nucleotide positions between OCT4A and PG1 are marked with an “∗.” The bottom row shows the sequences in which the common nucleotides in OCT4A were substituted with different bases. Left: Fluorescent images of FLAG immunostaining and co-transfection with RFP in both wild-type and mutant cells. Scale bars, 50 μm. Right: Relative positivity rate of FLAG-positive cells in mutant cells transfected with the vector or the full-length PG1 plasmid. The results for the wild type are shown in Figure 4H *n* = 5. Mean ± SD. Unpaired two-tailed Student’s *t* test. ∗ = *p* < 0.05, ∗∗ = *p* < 0.01, ∗∗∗ = *p* < 0.001, and ∗∗∗∗ = *p* < 0.0001; ns = not significant. (K) Fluorescence intensity of FLAG immunostaining following the forced expression of OCT4A wild-type and PG1 5′ UTR or its variants with different 5′ UTR lengths in the complementary region to OCT4A, transfected into NIH3T3 cells. *n* = 5. Mean ± SD. One-way ANOVA. ∗ = *p* < 0.05, ∗∗ = *p* < 0.01, ∗∗∗ = *p* < 0.001, and ∗∗∗∗ = *p* < 0.0001. (L) Fluorescence intensity per cell stained with the antibody C-10 against OCT4A. PA1 cells were transfected with three different amounts of synthesized RNA, each containing an equal number of RNA molecules for each type of synthesized RNA (Control RNA, PG1 5′ UTR wild, and PG1-5′ UTR -119 nt). Normalization was performed by dividing the number of DAPI-stained nuclei to account for differences in the number of cells per well. *n* = 3. Mean ± SD. One-way ANOVA. ∗ = *p* < 0.05, ∗∗ = *p* < 0.01, ∗∗∗ = *p* < 0.001, and ∗∗∗∗ = *p* < 0.0001. Plasmid vectors were introduced as mock controls in (A, H–K), and control RNA was used in (C–G, and L).

As PG1 is typically translated at very low levels, we investigated whether it functions primarily as a non-coding RNA. To examine its potential regulatory effect on its parental gene, OCT4A, we co-expressed OCT4A with an FLAG tag alongside either full-length PG1 or its untranslated regions (5′ UTR or 3′ UTR) in mouse fibroblast NIH3T3 cells. The results showed that the co-expression of full-length PG1 or its 5′ UTR significantly reduced OCT4A protein levels, whereas co-expression of the 3′ UTR had no effect (Figure 4A).

To determine whether PG1 affects endogenous OCT4A translation, we introduced a series of PG1 expression plasmids into PA1 cells known for their high OCT4A expression. Overexpression of full-length PG1 or its 5′ UTR alone resulted in a significant reduction in OCT4A protein levels, as detected using a monoclonal antibody specific to OCT4A (C-10; sc5279). Compared to the control, the expression of full-length PG1 or its 5′ UTR alone led to an approximately 50% decrease in OCT4 levels (Figure 4B). These results indicate that the 5′ UTR of PG1 suppresses the translation of its parental gene OCT4A.

To confirm whether PG1 functions as an RNA molecule, we synthesized RNA corresponding to constructs containing either the 5′ and 3′ UTR regions of PG1 or only the 5′ UTR region using T7 RNA polymerase *in vitro*. These RNA molecules were then introduced into PA1 cells, and OCT4A translation efficiency was assessed using an antibody against OCT4 and by measuring mean fluorescence intensity. RNA containing only the PG1 5′ UTR exhibited a mean fluorescence intensity of 4.5 × 10^4^ ± 4.5 × 10^3^ (SD), whereas the control group showed a significantly higher mean fluorescence intensity of 8.3 × 10^4^ ± 7.6 × 10^3^ (SD) (Figure 4C). Additionally, the expression levels of *OCT4A* mRNA remained unchanged in all samples, indicating that PG1 did not alter *OCT4A* mRNA levels (Figure 4D). Furthermore, western blot analysis using an OCT4A-specific antibody (C-10; sc5279) confirmed that the PG1 5′ UTR RNA suppresses the protein level of OCT4A, consistent with the results obtained from immunostaining (Figure 4E; uncropped images are shown in Figure S8). These results demonstrate that PG1, particularly its 5′ UTR, suppresses OCT4A translation at the post-transcriptional level.

Consistent with this translational regression, we observed a reduction in the expression of the undifferentiated stem cell marker alkaline phosphatase (ALP) (Figure 4F) and an increase in the expression of the differentiated marker nestin in PA1 cells (Figure 4G). Based on the results shown in Figures 4A–4G, we inferred that the 5′ UTR of PG1 suppresses OCT4A translation. To further investigate this mechanism, we generated constructs in which the PG1 5′ UTR was progressively deleted in approximately 60 bp increments from the 5′ end (designated as Δ1–Δ6) and examined their effects on translation. As a reporter gene, we co-transfected OCT4A containing both its 5′ UTR and 3′ UTR with an FLAG tag fused in-frame along with the PG1 constructs described above. OCT4A expression was detected using an anti-FLAG antibody. The results showed that OCT4A translation suppression was maintained up to Δ2; however, further deletions progressively reduced this effect (Figure 4H).

Given that the 5′ proximal 119 bp of the PG1 5′ UTR suppresses OCT4A translation, we investigated which region of OCT4A was affected by this suppression. Therefore, we generated OCT4A mutant DNA constructs lacking either the 5′ UTR or 3′ UTR and co-expressed them with full-length PG1 in NIH3T3 cells. Consequently, when the 5′ UTR of OCT4A was deleted, translational suppression by PG1 was not observed (Figure 4I).

To further analyze the interaction between the PG1 and 5′ UTR of OCT4A, we compared their sequences. This analysis revealed that the 5′ proximal region of the PG1 5′ UTR is complementary to the 3′ region of the OCT4A 5′ UTR, with approximately 50% sequence identity (Figure 4J, upper panel). Based on this finding, we introduced nucleotide substitutions into OCT4A to replace the homologous bases with non-complementary bases. Subsequent experiments showed that in these modified OCT4A constructs, PG1-mediated translational suppression was abolished (Figure 4J, lower panel).

Based on these findings, we hypothesized that OCT4A translation suppression could be induced solely by the 5′ proximal 119 bp of PG1’s 5′ UTR, which acts as a complementary regulatory element. To verify this, we examined the effect of introducing either the 5′ proximal 119 bp or the 5′ proximal 89 bp of the PG1 5′ UTR. When NIH3T3 cells were transfected with wild-type OCT4A and analyzed using the same detection method, both the 5′ proximal 119 bp and 89 bp of the PG1 5′ UTR exhibited a suppression effect equivalent to that of the full-length PG1 5′ UTR (Figure 4K). These results demonstrate that even the 5′ proximal 89 bp of the PG1 5′ UTR alone is sufficient to induce the translational suppression of OCT4A. Furthermore, we confirmed in PA1 cells that introducing the full-length and 5′ proximal 119 nt length regions of the PG1 5′ UTR as RNA exerted a similar suppressive effect on endogenous OCT4A (Figure 4L).

Additionally, the 42 bp region immediately upstream of the AUG start codon in the 5′ UTR sequences of OCT4A and PG1 is identical (Figure 3A). Therefore, we investigated whether the 5′ proximal region of PG1 could act complementarily to its own 5′ UTR and contribute to translational suppression. Using the 58bp 5′ UTR (Δ6)-PG1 tagged with FLAG at the C-terminus, we confirmed significant suppressive effects on translation (Figure S5B).

### PG1 undergoes rapid degradation and lacks tumorigenic and growth-promoting activity

**Figure 5:**
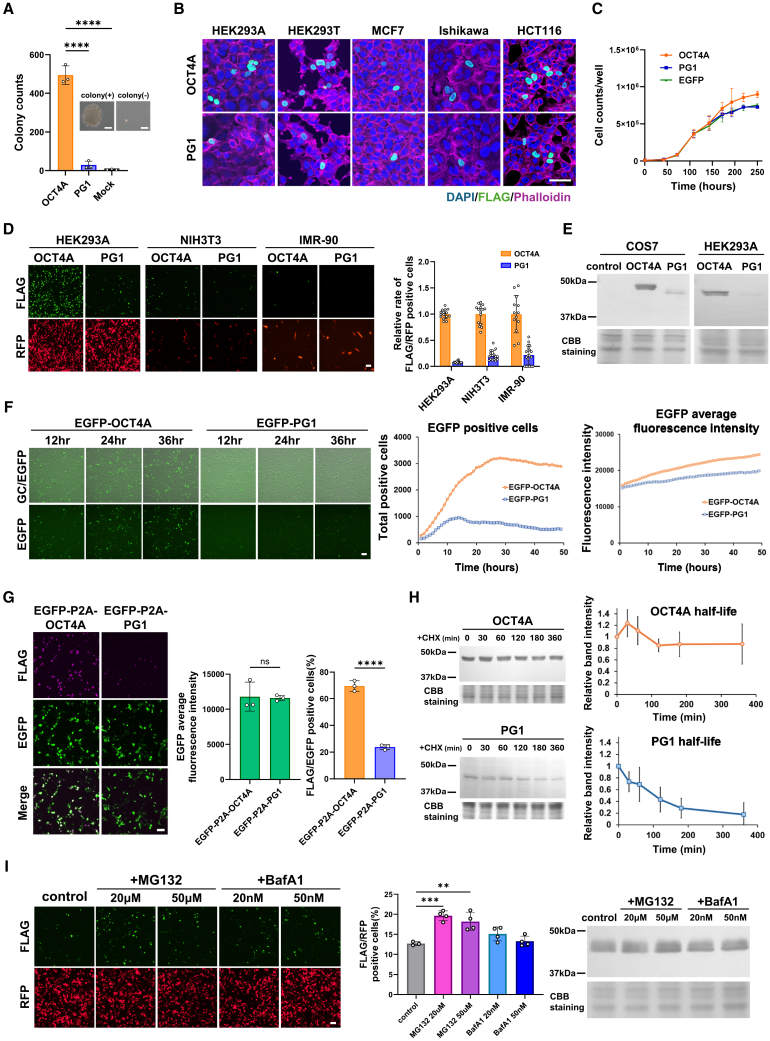
PG1 proteins are easily degraded and do not have tumorigenic or proliferative activity, unlike parent OCT4A (A) Tumorigenic activity of NIH3T3 cells was evaluated using a soft agar colony formation assay. *n* = 3. Mean ± SD. One-way ANOVA. ∗ = *p* < 0.05, ∗∗ = *p* < 0.01, ∗∗∗ = *p* < 0.001, and ∗∗∗∗ = *p* < 0.0001. Scale bars, 50 μm. (B) The intracellular localization of C-terminal FLAG-tagged PG1 and OCT4A was confirmed by immunostaining with an anti-FLAG antibody following forced expression of these proteins in HEK293A, HEK293T, MCF7, Ishikawa, or HCT116 cells. Scale bars, 50 μm. (C) Growth curves of stable mix lines stably expressing OCT4A or PG1 (and EGFP control) in HCT116 colon cancer cells. *n* = 3. Mean ± SD. (D) Immune positivity rates following forced expression of OCT4A and PG1 proteins with FLAG tags compared to RFP control. Left: Fluorescent antibody staining of FLAG and RFP in normal human and mouse cells (HEK293A, NIH3T3, IMR-90) with forced expression. Right: Relative percentage of FLAG-positive cells normalized to RFP transfection efficiency. *n* = 3. Mean ± SD. (E) Western blot analysis of OCT4A and PG1 expression in COS7 and HEK293A cells using anti-FLAG antibodies. An acrylamide gel stained with Coomassie Brilliant Blue (CBB) was used as the loading control. (F) Fluorescence intensity of cells expressing EGFP-fusion proteins. Left: Fluorescence images at 12, 24, and 36 h after HEK293A cell transfection. Right: Time-course analysis of fluorescence intensity using time-lapse microscopy. (G) Detection of EGFP and FLAG-tagged OCT4A and PG1 by the P2A-based polycistronic expression Left: Fluorescent antibody staining of FLAG and EGFP fluorescence images in HEK293A cells with forced expression. Middle: Average EGFP fluorescence intensity. Sixteen fields of view were captured per well, and the mean fluorescence intensity was determined. Right: Relative percentage of FLAG-positive cells normalized to EGFP transfection efficiency. *n* = 3. Mean ± SD. Unpaired two-tailed Student’s *t* test. ∗ = *p* < 0.05, ∗∗ = *p* < 0.01, ∗∗∗ = *p* < 0.001, and ∗∗∗∗ = *p* < 0.0001; ns = not significant. Scale bars, 50 μm. (H) Half-lives of OCT4A and PG1 proteins evaluated by western blotting in COS7 cells. CHX: cycloheximide (minutes after cycloheximide treatment). The acrylamide gel stained with CBB is shown under the panel as a loading control. *n* = 3. Mean ± SD. (I) Effects of the proteasome inhibitor MG132 and autophagy inhibitor bafilomycin A1 on PG1 stability in COS7 cells. Left: Immunostaining using FLAG-tagged fluorescent antibodies. RFP indicated that transfection was performed uniformly. Scale bars, 50 μm. Middle: Proportion of FLAG-positive cells among the transfected cells. *n* = 3. Mean ± SD. One-way ANOVA. ∗ = *p* < 0.05, ∗∗ = *p* < 0.01, ∗∗∗ = *p* < 0.001, and ∗∗∗∗ = *p* < 0.0001. Right: Quantification of PG1-FLAG protein levels by western blotting after varying the drug dose and in the presence or absence of treatment. An acrylamide gel stained with CBB was used as the loading control.

Our results suggest that PG1 is rarely translated under normal conditions but may be translated following 5′ UTR sequence deletions or mutations in diseases, such as cancer. To investigate whether PG1 is functional when produced, we conducted a detailed analysis of its stability and biological activity. To assess its tumorigenic potential, we forcibly expressed PG1 by introducing an ORF sequence fused with a FLAG tag at the C-terminus of NIH3T3 cells, a non-tumorigenic mouse fibroblast cell line. Unlike OCT4A, stable PG1 expression was insufficient to induce scaffold-independent growth in soft agar, a hallmark of cancer cells (Figure 5A). Similarly, in human normal fibroblast IMR-90 cells, neither OCT4A nor PG1 exhibited tumorigenic activity (data not shown). To determine its subcellular localization, we examined FLAG-tagged PG1 using immunofluorescence. PG1 was predominantly localized in the nucleus in all tested cell lines (Figure 5B).

Next, we evaluated whether PG1 influenced cell proliferation by generating a stable PG1-expressing HCT116 colorectal cancer cell line. PG1 expression did not promote proliferation (Figure 5C). Additionally, when PG1 was strongly expressed, its protein levels were markedly lower than those of OCT4A (Figures 5D and 5E; uncropped images are shown in Figure S8). Moreover, we generated and expressed EGFP-fusion proteins and monitored their expression using time-lapse microscopy. EGFP-PG1 fluorescence was significantly lower than that of EGFP-OCT4A at 12 h post-transfection, and its intensity did not increase over time (Figure 5F and Videos S1 and S2). Time-lapse imaging confirmed that the low PG1 signal was not due to cytotoxicity or cell death (Video S2).

Video S1. Live-cell imaging of HEK293A cells expressing EGFP-OCT4A fusion protein, related to Figure 5F The EGFP fluorescence signal (green) is overlaid with Gradient Contrast (GC) images to show the subcellular localization of the protein. Images were captured every 30 min. Scale bar represents 100 μm.

Video S2. Live-cell imaging of HEK293A cells expressing EGFP-PG1 fusion protein, related to Figure 5F The EGFP fluorescence signal (green) is overlaid with Gradient Contrast (GC) images to show the subcellular localization of the protein. Images were captured every 30 min. Scale bar represents 100 μm.

To compare the stability of OCT4A and PG1, we used 2A peptide (P2A)-based polycistronic expression plasmids (OCT4A-p2A-EGFP and PG1-p2A-EGFP). Although EGFP intensity appeared similar between the two constructs, FLAG tag staining revealed that PG1 protein expression was significantly lower than that of OCT4A (Figure 5G). Next, we investigated the half-life of the PG1 protein using a cycloheximide chase assay. The PG1 half-life was approximately 1.5 h, which was significantly shorter than that of OCT4A (approximately 6 h) (Figure 5H; uncropped images are shown in Figure S8). To determine the mechanism of PG1 degradation, we treated the cells with the proteasome inhibitor MG132 and the autophagy inhibitor Bafilomycin A1 (BafA1). MG132 treatment partially stabilized PG1, whereas BafA1 had no significant effect (Figure 5I; uncropped images are shown in Figure S8), suggesting that PG1 degradation is mediated via the proteasome pathway.

### PG1 protein stability and weak transcriptional activity depend on OCT4A or SOX2

Although the PG1 protein is highly susceptible to degradation, its sequence closely resembles that of the DNA-binding domain of OCT4A. To clarify its potential regulatory role, we re-examined the transcriptional activity of PG1 and its ability to interact with other transcriptional regulators, particularly OCT4A and SOX2.

**Figure 6:**
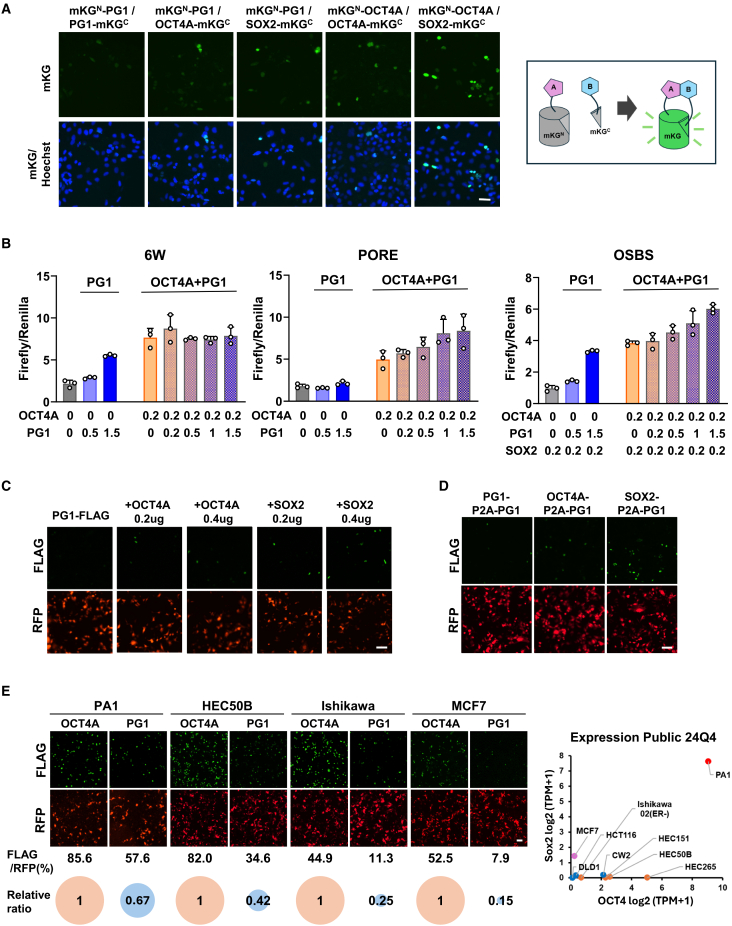
PG1 proteins can stabilize and exhibit transcriptional activity by dimer formation with OCT4A and SOX2 (A) Evaluation of OCT4A-OCT4A and PG1-PG1 homodimer formation, as well as PG1 heterodimerization ability with OCT4A and Sox2, using the coral hue assay in HEK293A cells. Scale bars, 50 μm. (B) Evaluation of the transcriptional regulatory activity of the co-expression of OCT4A and PG1 in the dual-luciferase assay when increasing the amount of PG1 in COS7 cells. *n* = 3. Mean ± SD. (C) Expression levels of PG1 protein when co-transfected with OCT4A or SOX2 in COS7 cells. Scale bars, 100 μm. The results of protein detection using western blotting are shown in Figure S6. (D) Expression level of PG1 protein in the presence of OCT4A or SOX2 using the P2A peptide for co-expression of the two genes in COS7 cells. Scale bars, 100 μm. The results of protein detection using western blotting are shown in Figure S6. (E) Left: Detection of forcibly expressed PG1 protein in various cell lines (PA1, HEC50B, Ishikawa, and MCF7) with different OCT4A expression levels. Scale bars, 100 μm. Right: Expression levels of OCT4A and SOX2 mRNA in the cell lines used in the CCLE database.

As OCT4A functions as a transcriptional regulator both as a monomer and through homodimerization or heterodimerization with SOX2,^32^^,^^33^^,^^34^ we first evaluated the dimerization potential of PG1 using a coral hue assay in HEK293A cells (Figure 6A). The results showed that PG1 formed heterodimers with OCT4A and SOX2, although the nuclear signal was weaker than that of OCT4A homodimers and OCT4A-SOX2 heterodimers. In contrast, only faint diffuse fluorescence was observed throughout the cytoplasm in cells expressing PG1 homodimers, indicating that PG1 does not stably exist as a homodimer, unlike OCT4A.

To assess the transcriptional regulatory activity of PG1, we performed a dual-luciferase assay using three sequences: 6W, PORE, and OSBS. These sequences correspond to OCT4A acting as a monomer, homodimer, or heterodimer with SOX2 (Figure 6B).^33^^,^^35^ In the 6W sequence assay, where OCT4A functions as a monomer, PG1 exhibited dose-dependent activity but was significantly less potent than OCT4A. In the PORE sequence, where OCT4A operates as a homodimer, PG1 showed no transcriptional activity, consistent with its inability to form stable homodimers. In the OSBS sequence assay, where OCT4A acts as a heterodimer with SOX2, PG1 displayed weak activity when introduced at high levels, although it remained significantly less active than OCT4A activity.

Next, we investigated the effect of PG1 when co-expressed with OCT4A. Increasing PG1 levels enhanced transcriptional activity at the PORE and OSBS sequences in a dose-dependent manner, whereas no enhancement was observed at the 6W sequence. PG1 did not inhibit the transcriptional regulatory activity of OCT4A under any of these conditions.

To investigate whether heterodimerization contributes to PG1 stabilization, we analyzed PG1 protein levels following co-expression with OCT4A or SOX2 using immunostaining and western blotting. The results showed that PG1 protein levels increased in a dose-dependent manner when co-expressed with OCT4A or SOX2 (Figures 6C and S6A). Using a P2A-linked construct that ensured equal expression of OCT4A and SOX2 within the same cells, we observed a clear increase in PG1 protein levels, particularly in the presence of SOX2 (Figures 6D and S6B).

Furthermore, we examined PG1 expression in teratocarcinoma, endometrial cancer, and breast cancer cell lines that endogenously express varying levels of OCT4A and/or SOX2 (Figure 6E). Higher endogenous expression levels of OCT4A or SOX2 corresponded to an increased detection of PG1-FLAG-positive cells.

### Role of Gln259 in the functional differences between OCT4A and PG1 proteins

**Figure 7:**
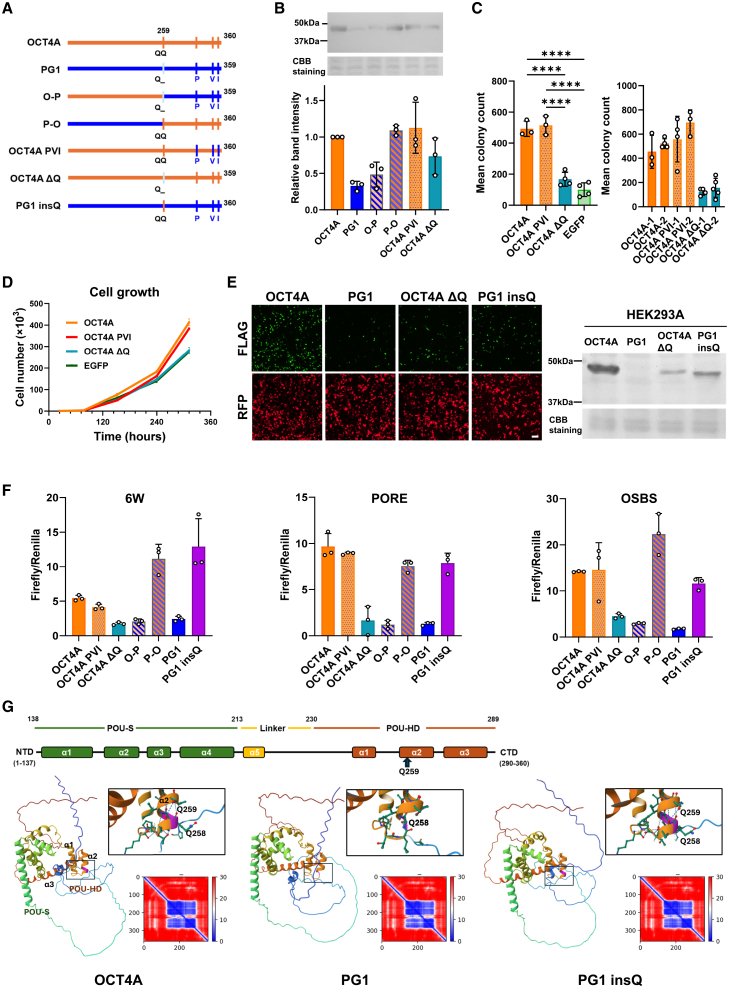
Gln259 is essential for OCT4A transformation, transcriptional regulatory activity, and protein stability (A) Schematic diagram of OCT4A, PG1OCT4A, and PG1 protein mutants with forced expression. All constructs contained a FLAG tag added at the C-terminus. (B) Western blot detection of OCT4A and PG1 protein mutants expressed in COS7 cells. The graph displays the quantification of Western blot band intensity. Band intensities were normalized against the total protein amount, as visualized by Coomassie Brilliant Blue (CBB) staining of the gel. *n* = 3. Mean ± SD. (C) Soft agar colony formation by cells stably expressing OCT4A and its mutants in NIH3T3 cells. Left: mixed stable expression lines; right: results of cloned stable expression for each line. n = 3–5. Mean ± SD. One-way ANOVA. ∗ = *p* < 0.05, ∗∗ = *p* < 0.01, ∗∗∗ = *p* < 0.001, and ∗∗∗∗ = *p* < 0.0001. (D) Growth curves for the stable expression of amino acid mutants OCT4A and PG1 in NIH3T3 cells. *n* = 3. Mean ± SD. (E) Immunofluorescence and western blotting revealed differences in the protein expression of the OCT4A, PG1, and Gln259 amino acid mutants in HEK293A cells. Scale bars, 100 μm. For western blotting, an acrylamide gel stained with CBB was used as the loading control. (F) Differences in the transcriptional regulatory activities of OCT4A and PG1 mutants in the dual-luciferase assay in COS7 cells. *n* = 3. Mean ± SD. (G) Impact of Gln259 on the predicted protein tertiary structure. We compared the predicted structures of OCT4, PG1, and PG1insQ using AlphaFold2. Upper: Indication of the protein domains and α-helix structures of OCT4A.The arrow indicates the position of Q259. (lower) plots represent the accuracy of the prediction model for each protein. The Q259 position is highlighted in pink.

As described above, the biological functions and stability of OCT4A and PG1 proteins differ significantly, despite their high amino acid sequence similarity (Figure S7). To determine which amino acid differences contribute to these functional disparities, we generated OCT4A and PG1 mutants, as shown in Figure 7A. First, we confirmed protein translation in various mutants using western blotting (Figure 7B). A comparison between the OCT4A-PG1 (O-P) and PG1-OCT4A (P-O) mutants indicated that the C-terminal region of PG1 (beyond amino acid position 259) was responsible for protein instability. Four amino acid differences were observed in this region (Figures 7A and S7). To further assess their impact, we generated two additional mutants, OCT4AΔQ (lacking Gln259) and a triple mutant OCT4A-PVI (carrying the L314P/A344V/T351I substitutions). Western blot analysis revealed that OCT4A-PVI exhibited protein levels comparable to those of OCT4A, whereas OCT4AΔQ exhibited significantly lower protein levels (Figure 7B; uncropped images are shown in Figure S9). To investigate whether Gln259 is involved in tumorigenesis and transcriptional regulation, we established stable NIH3T3 cell lines expressing OCT4A, OCT4A-PVI, OCT4AΔQ, or control EGFP and assessed their tumorigenic potential using soft agar colony formation assays. In both mixed and cloned stable expression cell lines, OCT4A-PVI exhibited tumorigenic potential similar to that of OCT4A, whereas OCT4AΔQ did not (Figure 7C). Growth curve analysis confirmed that OCT4A and OCT4A-PVI retained proliferative potential, whereas OCT4ΔQ did not (Figure 7D).

These findings strongly suggest that the presence of Gln259 is important for the differences between OCT4A and PG1. Therefore, we generated a mutant in which Gln259 was inserted into the PG1 protein (PG1insQ) and analyzed its protein stability. Immunofluorescence staining and western blotting in HEK293A cells revealed a marked improvement in the protein stability of PG1insQ (Figure 7E; uncropped images are shown in Figure S9). Next, we examined the transcriptional regulatory activities of OCT4A and PG1 mutants in COS7 cells using luciferase reporter constructs containing the 6W, PORE, and OSBS sequences. Both transcriptional regulatory activities were markedly reduced in OCT4AΔQ, whereas PG1insQ exhibited transcriptional regulation comparable to that of OCT4A (Figure 7F). Finally, we used AlphaFold2^36^ and Mol∗ Viewer^37^ to predict the 3D structures of OCT4A, PG1, and PG1insQ (Figure 7G).

## Discussion

In this study, we demonstrated that PG1 is not translated from its normal transcript but rather functions as an RNA molecule. Our findings indicate that the PG1 5′ UTR exerts a *cis*-acting effect that strongly suppresses translation. Furthermore, we identified a *trans*-acting suppressive effect of the PG1 5′ UTR, particularly at positions −424 to −336, on the translation of its parental gene OCT4A. Several mechanisms have been proposed for lncRNA-mediated translational suppression.^38^ Our findings suggest that a sequence of approximately 120 nucleotides within the PG1 5′ UTR, which is complementary to the region just upstream of the translation start site of OCT4A and PG1, may suppress translation by forming a secondary structure that hinders ribosome scanning, masks the start codon, or interferes with translation initiation factors. While previous studies have reported that pseudogenes can regulate the functions of their parental genes, the role of pseudogenes as non-coding RNAs has only recently gained attention.^3^^,^^39^^,^^40^ Our discovery that PG1, an RNA molecule, suppresses the translation of its parental gene, OCT4A, provides a fresh insight into the function of pseudogenes. These findings suggest the potential for developing RNA-based therapeutics that suppress OCT4 activity in CSCs.

The expression pattern of PG1 supports its functional significance in this context. Although PG1 mRNA expression is low in normal tissues, it is elevated in cancer tissues, suggesting a possible role in tumorigenesis. However, recent single-cell analyses have detected the co-expression of PG1 and stem cell factors at specific stages of early human embryogenesis.^41^^,^^42^ Our study demonstrated that PG1 suppressed OCT4A protein levels by approximately 50%, leading to a decrease in ALP activity and an increase in the expression of differentiation markers (Figures 4F and 4G). Given that OCT4A levels must be strictly regulated to maintain pluripotency,^43^ these findings suggest that PG1, as an RNA molecule, functions as an embryonic stage-specific factor that modulates stem cell states during early human development.

In cancer cells, PG1 may function in two distinct ways: as an RNA molecule and as a protein. Our results suggest that PG1 is rarely translated under normal conditions but may be translated following 5′ UTR sequence deletions or mutations in diseases such as cancer. Mutations in the PG1 5′ UTR have frequently been reported in cancer, supporting this possibility. Non-coding point mutations or indels may contribute to tumorigenesis by altering gene expression through mechanisms such as chromatin domain disruption, mRNA stability changes, and altered translation efficiency.^44^ When forcibly expressed, the PG1 protein undergoes active degradation after translation, suggesting that any translated PG1 protein is likely to have limited biological stability and is not functional.

Our quantitative analysis of OCT4A and PG1 mRNA expression indicated that OCT4A is associated with cancer malignancy, whereas PG1 showed no such association, at least in endometrial cancer, where PG1 gene amplification was not observed (Figures S1A and S1B). Moreover, PG1 expression was inversely correlated with cancer grade in both cell lines and clinical specimens (Figures 1 and 2). These results suggest that high PG1 mRNA expression, functioning as a lncRNA, may suppress OCT4A protein levels, potentially inhibiting malignant progression in low-grade endometrial cancer. This finding aligns with the notion that a higher proportion of OCT4A-positive cells within a cancer cell population correlates with increased malignancy, as demonstrated in our previous study.^19^ Additionally, consistent with our cell line data, OCT4 was highly expressed in endometrial cancers, whereas PG1 was predominantly expressed in colorectal cancers (Figure 1C), suggesting distinct tissue-specific roles for OCT4A and PG1.

OCT4A expression in endometrial cancer cell lines correlates with malignancy and is significantly associated with diabetes, obesity, and squamous differentiation (SQ) in clinical samples. Diabetes and obesity are well-established risk factors for endometrial cancer, and diabetic complications are correlated with poor prognosis.^45^^,^^46^ Histopathological findings of SQ in endometrial cancer indicate differentiation into benign or malignant squamous epithelium. A recent review suggested that SQ correlates with prognosis.^47^ From a cancer stem cell perspective, SQ may reflect plasticity within cancer cell populations, possibly driven by elevated OCT4A expression levels. Although not statistically significant, OCT4A expression also showed trends of association with tumor grade, MI+, LVSI+, and MELF+, all of which are recognized recurrence risk factors. Although large-scale studies are needed, these findings support the potential of OCT4A as a diagnostic and prognostic marker for early-stage endometrial cancer.

Our previous study demonstrated that high OCT4A expression correlates with poor prognosis in lung cancer and drug resistance in multiple cancer cell lines.^19^^,^^25^ Furthermore, we confirmed that there was no correlation between the expression patterns of OCT4A and PG1 in lung tumor samples (data not shown). These results suggest that OCT4A expression, but not PG1 expression, may indicate malignancy in somatic cancers lacking PG1 gene amplification or mutation.

Conversely, in gastrointestinal cancers frequently exhibiting PG1 gene amplification (Figure S1),^48^ genomic alterations such as translocation, mutation, or deletion in the 5′ UTR may change its function from suppressing OCT4A as a non-coding RNA to enhancing OCT4A activity as a protein. In contrast to OCT4A, PG1 expression did not promote cell proliferation (Figure 5C), contrary to the findings of a recent study.^22^ This finding indicates that PG1 does not function as an oncogene. In contrast to a recent report,^22^ our findings indicate that PG1 is almost exclusively localized in the nucleus of all the tested cell lines (Figure 5B).

Remarkably, we identified that a single amino acid difference at Gln259 significantly impacted the structure, stability, and function of PG1, preventing it from exhibiting OCT4A-like tumorigenic and transcriptional activity. Gln259 is conserved across mammalian OCT4 proteins (Table S2), highlighting its significance in evolution. Gln259 resides in the α-helical structure within the POU homeodomain, and its absence significantly alters the surrounding conformation (Figure 7G), potentially affecting protein stability. These findings strongly suggest that Gln259 is essential for the functional divergence between OCT4A and PG1. Our results indicate that PG1 lacks independent transcriptional activity but can weakly enhance the function of OCT4A by forming heterodimers with OCT4A and SOX2. Additionally, these results revealed that if the putative PG1 protein is translated into stem cells expressing OCT4A or SOX2, it can stabilize and exert weak transcriptional regulatory activity by forming heterodimers with partner proteins such as OCT4A and SOX2. Thus, even if PG1 protein is produced in cancer cells, it does not function autonomously; rather, it enhances OCT4A activity, potentially contributing to a worse prognosis in gastrointestinal cancers.

Regardless of any potential, yet unproven, protein product from PG1, our findings strongly emphasize that the primary role of PG1 in modulating OCT4A function is exerted by its RNA molecule. Specifically, PG1 acts as a non-coding RNA (ncRNA) that directly suppresses the translation of its parental gene, OCT4A. This finding is particularly intriguing, given the evolutionary trajectory of PG1. PG1 is absent in rodents and is thought to have originated specifically within the lineage leading to great apes.^22^ The antisense activity we identified, which is responsible for translational suppression, appears to be a unique feature of PG1 among the pseudogenes studied. We analyzed the upstream regions of related pseudogenes, such as PG3 and PG4, but found no sequences corresponding to the active antisense region identified in PG1. This lineage-specific emergence coupled with a unique regulatory mechanism suggests that the translational control we uncovered is specific to PG1, which may, in turn, explain its unique biological role and potential contribution to primate-specific evolution compared to other related pseudogenes.

Given the emergence of PG1 after the great ape divergence and its reported expression during early human embryogenesis,^41^^,^^42^ we propose that PG1 may introduce an additional layer of post-transcriptional regulation of OCT4A. PG1-mediated suppression of OCT4A translation may serve as a mechanism to fine-tune the balance between stem cell proliferation and differentiation during human development.

Overall, these results indicate that PG1 plays a multifaceted role in modulating OCT4A function. This study provides significant insights into OCT4A biology, the evolutionary emergence and lineage-specific function of PG1, and its functional relevance in human CSCs. Furthermore, the pinpointed region within the 5′ UTR of PG1 RNA identified in this study has the potential to suppress malignant progression driven by OCT4A. Conducting large-scale, accurate expression analyses across various cancers and investigating the inhibitory effects on malignant progression using patient-derived tumor models could be valuable for future cancer treatment strategies.

### Limitations of the study

First, while our *in vitro* data strongly support the function of PG1 as a non-coding RNA, the precise role of PG1 expression *in vivo* and its functional relevance to human physiology remain to be fully elucidated. We could not verify the presence or function of PG1 expression in early human embryos or its regulation of OCT4 in that context. Investigating the potential pleiotropic roles of PG1 in cancer cells and early development is a worthwhile future endeavor.

Second, the clinical and prognostic significance of PG1-mediated OCT4A regulation requires further validation. While our initial findings are promising, a major limitation of this study is the relatively small sample size used in the clinical analysis. Therefore, future studies must involve large-scale validation cohorts of patient-derived tumor samples and corresponding clinical data to confirm OCT4A expression as a reliable prognostic biomarker for early-stage endometrial cancer. Expanding our analysis to other cancer types will help clarify the broader significance of PG1-mediated OCT4A regulation in cancer.

## Resource availability

### Lead contact

Further information and requests for resources and reagents should be directed to and will be fulfilled by the Lead Contact, Mitsuko Kosaka (m-kosaka@md.okayama-u.ac.jp).

### Materials availability

The plasmids generated in this study are available upon reasonable request.

### Data and code availability

The authors declare that the data supporting the findings of this study are included in the article and its supplemental information files. The original, uncropped images of the Western blots and Coomassie Brilliant Blue (CBB)-stained gels have been deposited in Mendeley Data and are publicly available at https://doi.org/10.17632/dmk74k7y5v.1. Any additional information required to reanalyze the data reported in this article is available from the lead contact upon request No custom code was generated in this study.

## Acknowledgments

We thank the members of our laboratory for their helpful support. This study was supported by the Okayama University Hospital Biobank. The authors are grateful to Prof. Hiroyuki Yanai from the Okayama University Hospital for his helpful comments regarding the pathological and clinical data analysis.

This work was supported by JSPS KAKENHI Grant Numbers JP15K15016 and JP19K09287, and the Translational Research Network Program from the AMED (AMED Grant Number: JP16lm0103011j0003 and JP17lm0203008j0001) to M.K. This work was also supported by JSPS KAKENHI Grant Number JP23KJ1605 (to K.I.), and JST 10.13039/501100025019SPRING Grant Number: JPMJSP2126 (to K.I. and S.M.N.O.). Additionally, K.I. was a recipient of a JSPS Research Fellowship for Young Scientists (DC2).

Sample usage was supported by AMED (Genomic medicine R&D platform utilization system via Platform Program for Promotion of Genome Medicine on Biobank—construction and utilization biobank for genomic medicine realization; B-Cure [JP21km0405401]).

## Author contributions

K.I.: collection and/or assembly of data, data analysis and interpretation, article writing, and final approval of article. M.K.: conception and design, financial support, collection and/or assembly of data, data analysis and interpretation, article writing, and final approval of article. N.M.: conception and design, collection and/or assembly of data, data analysis and interpretation, article writing, and final approval of article. R.O.: collection and/or assembly of data and final approval of article. Y.N.: collection and/or assembly of data and final approval of article. S. M.N.O.: collection and/or assembly of data and final approval of article. H.M.: administrative support, provision of study material or patients, and final approval of article. A.K.: financial support, administrative support, and final approval of article.

## Declaration of interests

The authors declare no competing interests.

## Declaration of generative AI and AI-assisted technologies in the writing process

AlphaFold2 was used to generate the predicted protein structure shown in Figure 7G.

## STAR★Methods

### Key resources table

**Table:** 

REAGENT or RESOURCE	SOURCE	IDENTIFIER
**Antibodies**		

Mouse monoclonal anti-FLAG; clone M2	Sigma	Cat#F1804; RRID:AB_262044
Mouse monoclonal anti-OCT3/4; C-10	Santa Cruz	Cat #sc-5279; RRID:AB_628051
Rabbit anti-Human Nestin; N1602	Immuno-Biological Laboratories	Cat #18741; RRID: AB_494610
Alexa 488-conjugated goat anti-mouse IgG	Invitrogen	Cat #A-11029; RRID:AB_2534088
Alexa 594-conjugated goat anti-mouse IgG,	Invitrogen	Cat #A-11032; RRID:AB_2534091
Alexa 488-conjugated goat anti-rabbit IgG	Invitrogen	Cat #A-11034; RRID:AB_2576217
Mouse monoclonal alkaline phosphatase-conjugated anti-FLAG	Sigma-Aldrich	Cat #A9469; RRID:AB_439699

**Chemicals, peptides, and recombinant proteins**		

Dulbecco’s modified Eagle medium	FUJIFILM Wako	#041-30085
fetal bovine serum	Vitromex	#VM S1500, Batch No.F000210802
DIFCO™ Agar Noble	BD	#214220
Lipofectamine® 2000	Thermo Fisher Scientific	#11668019
Lipofectamine® 3000	Thermo Fisher Scientific	#L3000015
Hoechst	DOJINDO	#H342
DAPI	Invitrogen	#D1306
MG132	FUJIFILM Wako	#135-18453
Bafilomycin A1	CST	#54645
Cycloheximide	FUJIFILM Wako	#037-20991
NBT/BCIP Stock Solution	Roche	#11681451001
Phalloidin	Invitrogen	#A12380
ISOGENE II	Nippon Gene	#311-07361
DNase I	Invitrogen	#55804
KAPA SYBR FAST Universal 2× qPCR Master Mix	Kapa Biosystems	#KK4600

**Critical commercial assays**		

Neon Transfection System	Thermo Fisher Scientific	#MPK10025
Dual-Luciferase® Kit	Promega	#E1910
PrimeScript II first-strand cDNA synthesis kit	Takara	#6210A/B

**Deposited data**		

Cancer Cell Line Encyclopedia (CCLE) Expression Public 24Q4 database	Broad Institute	https://portals.broadinstitute.org/ccle
Endometrial and colorectal cancer RNAseq dataset	Halling K, Diguardo M (Mayo Clinic)	GSE146889
UniProtKB/TrEMBL	European Bioinformatics Institute	https://www.uniprot.org/
The original, uncropped images of the Western blots and Coomassie Brilliant Blue-stained gels	Mendeley Data	https://doi.org/10.17632/dmk74k7y5v.1

**Experimental models: Cell lines**		

Ishikawa	ECACC	ECACC 99040201; RRID:CVCL_2529
MCF7	JCRB	JCRB0134; RRID:CVCL_0031
HEC50B	JCRB	JCRB1145; RRID:CVCL_2929
HEC265	JCRB	JCRB1142; RRID:CVCL_2928
HEC151	JCRB	JCRB1122; RRID:CVCL_2925
DLD-1	JCRB	JCRB9094; RRID:CVCL_0248
HCT116	RIKEN BRC	RCB2979; RRID:CVCL_0291
NIH3T3	RIKEN BRC	RCB2767; RRID:CVCL_0594
IMR-90	JCRB	JCRB9054; RRID:CVCL_0347
PA1	JCRB	JCRB9061; RRID:CVCL_0479
COS7	RIKEN BRC	RCB0539; RRID:CVCL_0224
HEK293A	Thermo Fisher Scientific Inc.	R70507; RRID:CVCL_6910
HEK293T	Dr. Hiroshi Sasaki (Osaka University)	RRID:CVCL_0063

**Oligonucleotides**		

See Figure S1 for list of RT-qPCR primers	–	–

**Recombinant DNA**		

See supplemental information (Figures S10 and S11) for list of recombinant plasmid DNA	–	–

**Software and algorithms**		

OLYMPUS cell Sens APEX4.2	EVIDENT/OLYMPUS	–
GEO2R	NCBI	https://www.ncbi.nlm.nih.gov/geo/geo2r/
galaxy	The Galaxy Community	https://usegalaxy.org/
Integrative Genomics Viewer (IGV) Version 2.16.2	Robinson, J., Thorvaldsdóttir, H., Winckler, W. et al.^48^	https://igv.org/
ImageJ	NIH, Bethesda,MD	https://imagej.net/ij/
GraphPad Prism10 version 10.2.3	Graphpad	https://www.graphpad.com/
AlphaFold2	Jumper et al.^36^	https://alphafold.ebi.ac.uk/
Mol∗ Viewer	Sehnal, David et al.	https://molstar.org/viewer/

### Experimental model and study participant details

#### Human participants

Endometrial endometrioid carcinoma patient samples (n=61) were collected from the Okayama University Hospital Biobank (Okadai Biobank; Ref. No. OC22006). All patients with endometrial cancer were diagnosed with stage I disease. Tumor tissues were collected during surgical resections (2017–2021) and snap-frozen at -80°C or stored in RNA later solution (Qiagen) until RNA extraction.

Written informed consent was obtained from all participants prior to their operation. All procedures were performed in accordance with the Declaration of Helsinki. Clinicopathological data for each patient were retrospectively obtained from the medical records of Okayama University Hospital. The clinical and histopathological characteristics of the patients are described in Table S1. This study (No. K2112–032) was approved by the Ethics Committee of the Okayama University Graduate School of Medicine, Dentistry, and Pharmaceutical Sciences and Okayama University Hospital (Okayama, Japan).

As this study focuses on endometrial cancer, only female subjects were included. Accordingly, the influence of sex and/or gender was not assessed as all subjects were of the same biological sex.

#### Cell lines

The Ishikawa (ECACC99040201), MCF7 (JCRB0134), HEC50B (JCRB1145), HEC265 (JCRB1142), HEC151 (JCRB1122), DLD-1 (JCRB9094), HCT116 (RCB2979), NIH3T3 (RCB2767), IMR-90 (JCRB9054), PA1 (JCRB9061) and COS7 (RCB0539) cell lines were sourced from established cell banks—JCRB (Osaka, Japan), RIKEN BRC (Tsukuba, Japan), or ECACC (Salisbury, U.K.)—in 2016 or later and were passaged for fewer than 6 months prior to experimentation. HEK293A cells were purchased from Thermo Fisher Scientific. HEK293T cell lines were generously provided by Dr. Hiroshi Sasaki (Osaka University). RNA extraction from these cell lines was performed within 2 weeks of receipt and did not exceed three passages. The cell cultures were maintained at 37°C in a 5% CO2 incubator. For monolayer culture, cells were maintained in culture dishes or plates (Sumitomo Bakelite) in Dulbecco’s modified Eagle medium (DMEM) supplemented with 8% fetal bovine serum. All cell lines used in this study were routinely tested for mycoplasma contamination.

### Method details

#### Cell culture, imaging and gene transfection

For gene transfection into various cell lines, Lipofectamine® 2000 or Lipofectamine® 3000 (Thermo Fisher Scientific) was utilized according to the manufacturer’s instructions. For cell lines with low transfection efficiency using Lipofectamine 3000 (such as IMR-90), the Neon Transfection System (Thermo Fisher Scientific) was used. The DNA constructs generated for this study are described in detail in the supplemental information (Figures S10 and S11). Hoechst (H342-Cellstain®- Hoechst 33342 solution; DOJINDO) was used as a nuclear marker when required. Cell images were acquired using the APX-100 Digital Imaging System (EVIDENT/OLYMPUS) and subsequently processed and analyzed using the OLYMPUS cellSens APEX4.2 software. Bright-field images were observed using either phase contrast or Gradient Contrast (GC) techniques. Time-lapse imaging was performed using the APX-100 digital imaging system with a TOKAI HIT STXG-APX-SET.

#### Soft agar colony formation assay

The anchorage-independent growth of cells was evaluated using a soft agar colony formation assay, performed as previously described^19^ with minor modifications. The assay was performed in 6 cm dishes using Difco™ Agar Noble (BD Biosciences). To prepare the bottom (base) layer, a 1.4% (w/v) agar solution in culture medium was prepared, and 2 mL of this mixture was added to each dish and allowed to solidify at room temperature. For the top (cell) layer, cells were suspended in a 0.7% (w/v) agar solution at a density of 2×10^3^ cells per dish. 3 mL of this cell-agar suspension was gently layered onto the solidified bottom layer. After the top layer had solidified, 1 mL of fresh culture medium was added to the top to prevent desiccation. The dishes were incubated at 37°C in a 5% CO_2_ atmosphere for 18 days. Colonies were monitored and then counted by visual inspection. Each experiment was performed in triplicate.

#### Dual-luciferase assay

Each DNA construct or empty pGL-4.1 with the phRG-TK plasmid (Promega) was transiently co-transfected into COS7 and other cells using Lipofectamine® 2000 or Lipofectamine® 3000 (Thermo Fisher Scientific), according to the manufacturer’s protocol. After an additional 30 h of incubation, cell lysates were prepared, and luciferase activity was assayed using the Dual-Luciferase® Kit (Promega) in a FlexStation 3 Multi-Mode Microplate Reader (Molecular Devices, courtesy of the Central Research Laboratory, Okayama University Medical School). We standardized the firefly luciferase activity, which is presented as relative values, to that of Renilla luciferase.

#### Western blot

Cells were transfected with the indicated DNA constructs and/or RNA using Lipofectamine® 3000 (Invitrogen) according to the manufacturer’s instructions. In the experiment (Figure 5I), following 32 to 48 h of transfection, a subset of the cells was treated with the proteasome inhibitor MG132 (20 μM or 50 μM; #135-18453; FUJIFILM Wako) or the lysosomal inhibitor bafilomycin A1 (#54645; CST) for 4 h. Control samples were treated with an equivalent volume of vehicle (e.g., DMSO). Subsequently, all cells were washed with Phosphate-Buffered Saline (PBS) and lysed for SDS-Polyacrylamide Gel Electrophoresis (SDS-PAGE).

Protein samples were separated by 10.5% SDS-PAGE and subsequently transferred onto polyvinylidene fluoride membranes (#IPVH00010; Merck Millipore). Membranes were blocked for 1 h with 2% skimmed milk in Tris-Buffered Saline containing 0.1% Tween-20 (TBST). The membranes were then probed with primary antibodies: mouse monoclonal alkaline phosphatase-conjugated anti-FLAG antibody (clone M2; #A9469; Sigma-Aldrich) or mouse monoclonal anti-OCT3/4 antibody (1/200; C-10; #sc-5279, Santa Cruz). For the anti-OCT3/4 primary antibody, detection was conducted using an anti-mouse IgG AP conjugate secondary antibody (1/2500; #S372B; Promega). Finally, the membranes were developed using NBT/BCIP Stock Solution (#11681451001; Roche). Uncropped images of Western blot membranes and CBB-stained gels are presented in Figures S8 and S9, and the original whole data have been deposited in Mendeley Data (https://doi.org/10.17632/dmk74k7y5v.1).

#### Immunocytochemistry

Cells were transfected with each DNA construct using Lipofectamine® 3000, if required. The cells were fixed with 4% paraformaldehyde for 15 min at 20–25°C. The cells were then permeabilized with 0.2% Triton X-100 for 15 min. Mouse monoclonal anti-FLAG antibody (1/500; clone M2; #F1804, Sigma), mouse monoclonal anti-OCT3/4 antibody (1/100; C-10; #sc-5279, Santa Cruz), and rabbit anti-human Nestin antibody (1/100; N1602; #18741, Immuno-Biological Laboratories) were used as primary antibodies. The cells were incubated with primary antibodies for 45 min at room temperature and washed thrice with PBS. The cells were incubated with secondary antibodies (1/1000; Alexa 488-conjugated goat anti-mouse IgG, #A-11029; Invitrogen), (1/1000; Alexa 594-conjugated goat anti-mouse IgG, #A-11032; Invitrogen), and (1/1000; Alexa 488-conjugated goat anti-rabbit IgG, #A-11034; Invitrogen). The cells were then counterstained with DAPI (#D1306; Invitrogen). Phalloidin (A12380; Invitrogen) was used to visualize the cytoskeleton. In the negative controls, the primary antibody was replaced with PBS.

#### Detection of alkaline phosphatase activity

The cells were fixed with 4% paraformaldehyde for 15 min at room temperature. After incubation in 0.1 M Tris-HCl (pH 9.5) and 0.1 M NaCl for 5 min, the reaction was conducted at room temperature (20–25°C) for 30 min using NBT/BCIP Stock Solution (#11681451001; Roche).

#### *In vitro* RNA synthesis

A plasmid was constructed by inserting a sequence containing either the 5′ and 3′ UTRs of PG1 or only the 5′ UTR immediately downstream of the T7 promoter sequence. The plasmid was linearized with EcoRI and used as a template for *in vitro* transcription using T7 RNA polymerase (Takara). As a control, a construct in which the NanoLuc (Promega) sequence was inserted in the reverse orientation was used for the experiments. Lipofectamine 3000 was used to introduce synthetic RNA into the cells. was applied at final concentrations of 5 nM or 10 nM. To ensure that equal molar amounts were delivered across all experimental groups, the amount of RNA used for transfection was calculated based on the molecular weight of each transcript.

#### Total RNA extraction and cDNA preparation

Total RNA from each cell line and fresh tissue was extracted using ISOGENE II (Nippon Gene). Fresh tissues were immediately homogenized using a gentleMACS™ Octo Dissociator with Heaters (Miltenyi Biotec) and processed according to the manufacturer’s instructions. cDNA was synthesized from 0.5 μg RNA using oligo-dT primers and a PrimeScript II First-Strand cDNA Synthesis Kit (#6210A/B; Takara) according to the manufacturer’s instructions. Reverse transcriptase negative controls were used to assess genomic DNA contamination. DNase treatment (DNase I 1U/μL #55804; Invitrogen) was used only to quantify the amount of mRNA of exogenous genes transfected with plasmid DNA.

#### Real-time quantitative PCR (qPCR)

Triplicates of each sample were assayed using relative quantitative polymerase chain reaction (PCR) (KAPA SYBR FAST Universal 2× qPCR Master Mix; Kapa Biosystems) on a Step One Plus real-time PCR system (Thermo Fisher Scientific) (Central Research Laboratory, Okayama University Medical School) according to the manufacturer’s protocol. PCR amplification conditions were as follows: 95°C for 3 min, followed by 40 cycles of 95°C for 10 s and 60°C for 30 s, and melting curve analysis (60–95°C with a heating rate of 0.3°C/s and continuous fluorescence measurement). The levels of OCT4A and PG1 messages were quantified compared to mRNA encoding the hypoxanthine phosphoribosyl transferase (HPRT1) protein, as an internal reference. Data were analyzed using a threshold set in the linear range of amplification. The cycle number at which any sample crossed that threshold (Ct) was used to determine the fold difference. The fold difference was calculated as 2^−ΔCt^; ΔCt = [Ct (OCT4A or PG1) − Ct (HPRT1)]. Information on these PCR primer sets is described in Figure S1.

#### Gene expression analysis using public database

The mRNA expression matrix of the cell lines was obtained from the Cancer Cell Line Encyclopedia (CCLE) Expression Public 24Q4 database (https://portals.broadinstitute.org/ccle).^26^ The mRNA expression matrix in endometrial cancer and normal tissues in GSE146889 was extracted from the Gene Expression Omnibus (GEO) and analyzed using GEO2R.^28^ Sequencing data extracted from GEO (GSM440899 and GSM4408870) were mapped to reference genome files (GRCh38/hg38) using galaxy (https://usegalaxy.org/) through the process of FASTQC, Trimmomatic, HISAT2, Samtools view and sort, and The Integrative Genomics Viewer (IGV) to confirm mapping data and Sashimi plot.^49^

#### Image analysis

Images of intracellular fluorescence were acquired using the APX-100 Digital Imaging System (EVIDENT/OLYMPUS) or a Confocal Laser Scanning Microscope (FLUOVIEW FV3000). For quantitative analysis, more than 12 non-overlapping fields per well were captured at 10× magnification using APX-100. Subsequent positive cell detection, cell counting, and fluorescence intensity measurements were performed using OLYMPUS CellSens APEX 4.2 software. Densitometric quantification of western blotting images was performed using ImageJ software (1.53q; (NIH, Bethesda, MD, USA). The intensity of each protein band was calculated by normalizing against the total protein load visualized by CBB staining of the gel, which served as a loading control.

### Quantification and statistical analyses

Data are presented as mean ± standard deviation (SD) of at least three independent experiments. Depending on whether the distribution was normally distributed or not, statistical evaluation was conducted using two-tailed t-tests or one-way ANOVA tests and Mann–Whitney or Kruskal–Wallis tests. Differences with *p* < 0.05 were considered statistically significant. ∗ = *p* < 0.05; ∗∗ = *p* < 0.01; ∗∗∗ = *p* < 0.001; ∗∗∗∗ = *p* < 0.0001; ns = not significant. GraphPad Prism10 (version 10.2.3) was used for statistical analyses.
