# Supplementary material for: Multifaceted role of POU5F1P1 in regulating its parental stem cell gene, POU5F1
Source: iScience. 2026 Feb 27;29(4):115137. doi: 10.1016/j.isci.2026.115137 (PMC12995708; doi:10.1016/j.isci.2026.115137)
Supplement: Document S1. Figures S1–S11 and Tables S1 and S2 [file mmc1.pdf]

iScience, Volume *29*

Supplemental information

Multifaceted role of *POU5F1P1* in regulating its parental stem cell gene, *POU5F1*

Kyohei Irie, Mitsuko Kosaka, Nobuhiko Mizuno, Ryo Omae, Yoshimasa Nakatani, Sandi Myat Noe Oo, Hisashi Masuyama, and Ayano Kawaguchi

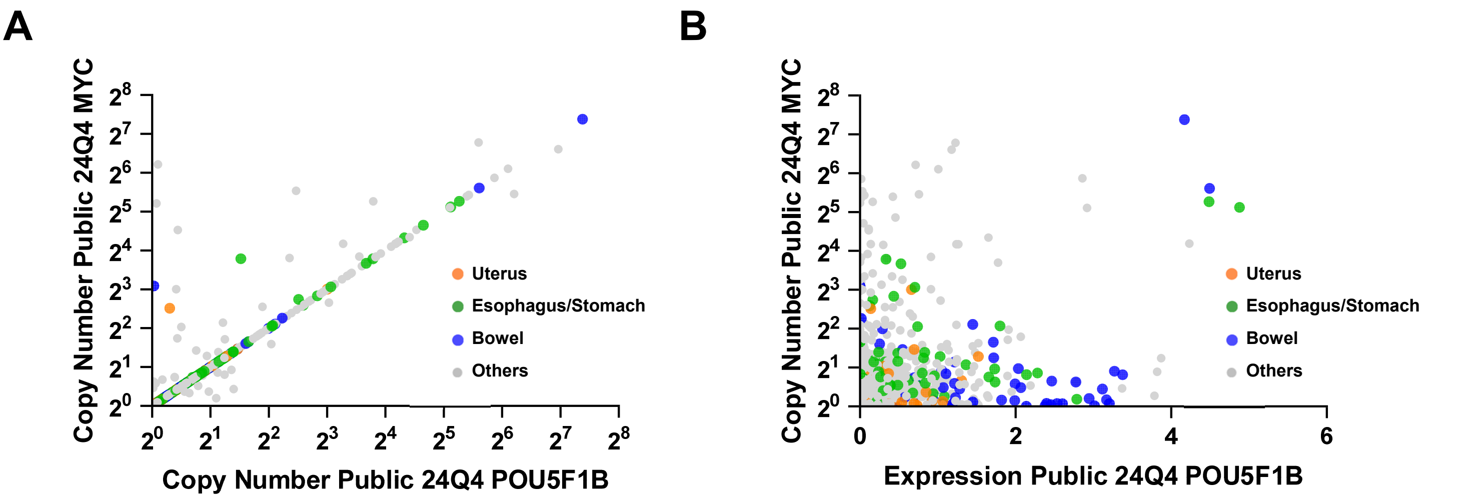

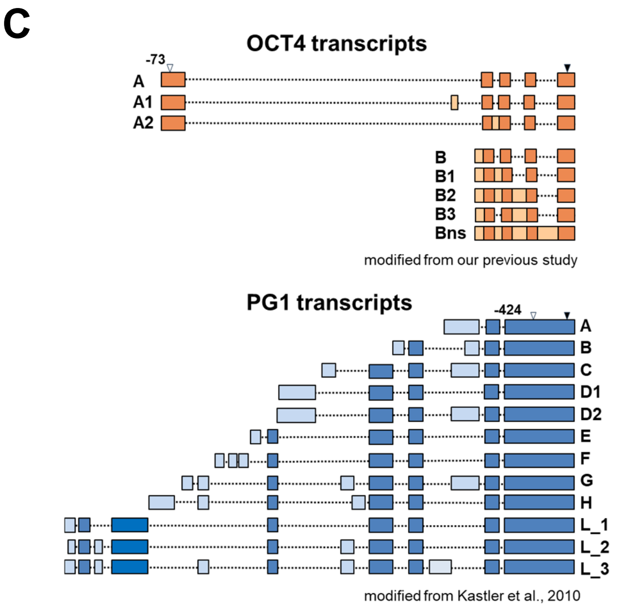

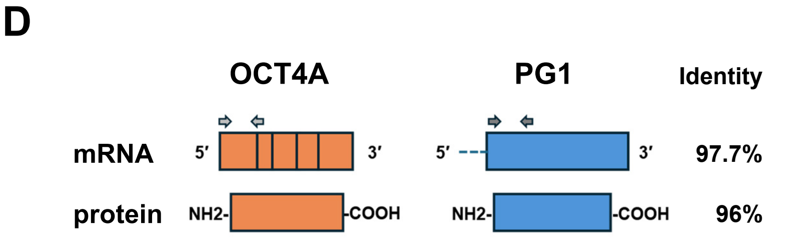

E

		Primer sequences for real-time PCR	product size
OCT4A	FO	5'-AGAGAGGGGTTGAGTAGTCCCTTCGCA-3'	496bp
	RV	5'-AGCTTTGATGTCCTGGGACTCCTCCGG-3'	
PG1	FO	5'-TCAGGCACTGTGTTCATTGCTAGTGAG-3'	549bp
	RV	5'-AGCTTTGATGTCCTGGGACTCCTCCGG-3'	
HPRT1	FO	5'-CATTATGCTGAGGATTTGGAAAGG-3'	129bp
	RV	5'-CTTGAGCACACAGAGGGCTACA-3'	

Figure S1. Molecular characterization of genes OCT4 and POU5F1B (PG1), including transcript-specific primer design and supplementary CCLE data for genes PG1 and MYC, related to Figure 1.

A) Correlation between the copy numbers of MYC and PG1. Data were obtained from cancer cell lines in the Cancer Cell Line Encyclopedia (CCLE) Public 24Q4 dataset. The Pearson correlation coefficient between the two is 0.85, indicating a strong correlation, as they are located on the same chromosome.

B) Correlation between MYC copy number and PG1 expression levels. Based on the CCLE Public 24Q4 dataset, MYC copy number was markedly elevated in gastrointestinal cell lines exhibiting high PG1 expression. In contrast, MYC copy number amplification was rarely observed in endometrial cancer cell lines.

C) Illustration of the multiple transcript variants of OCT4 and PG1, which were generated based on description and findings reported in the literature [19, 31]. The boxes represent exon regions, and the light-colored boxes indicate additional exons. Empty and solid triangles indicate the positions of the first AUG codon and termination codon, respectively.

D) Schematic representation of OCT4A and PG1 mRNA and proteins. The positions corresponding to the specific primers used in this study are indicated by arrows. The amino acid sequence identity between OCT4A and PG1 proteins is 96%. The mRNA sequence homology between the coding sequences of OCT4A and PG1 is 97.7%.

E) Primer sequences and product sizes used in real-time RT-PCR. The primers for PG1 cannot exclude genomic sequences, but it was confirmed that no product was generated in the absence of reverse transcriptase. The primer sequences for human HPRT1 (NM_000194) were custom synthesized based on the design provided by OriGene Technologies, Inc. (Catalog No. HP200179).

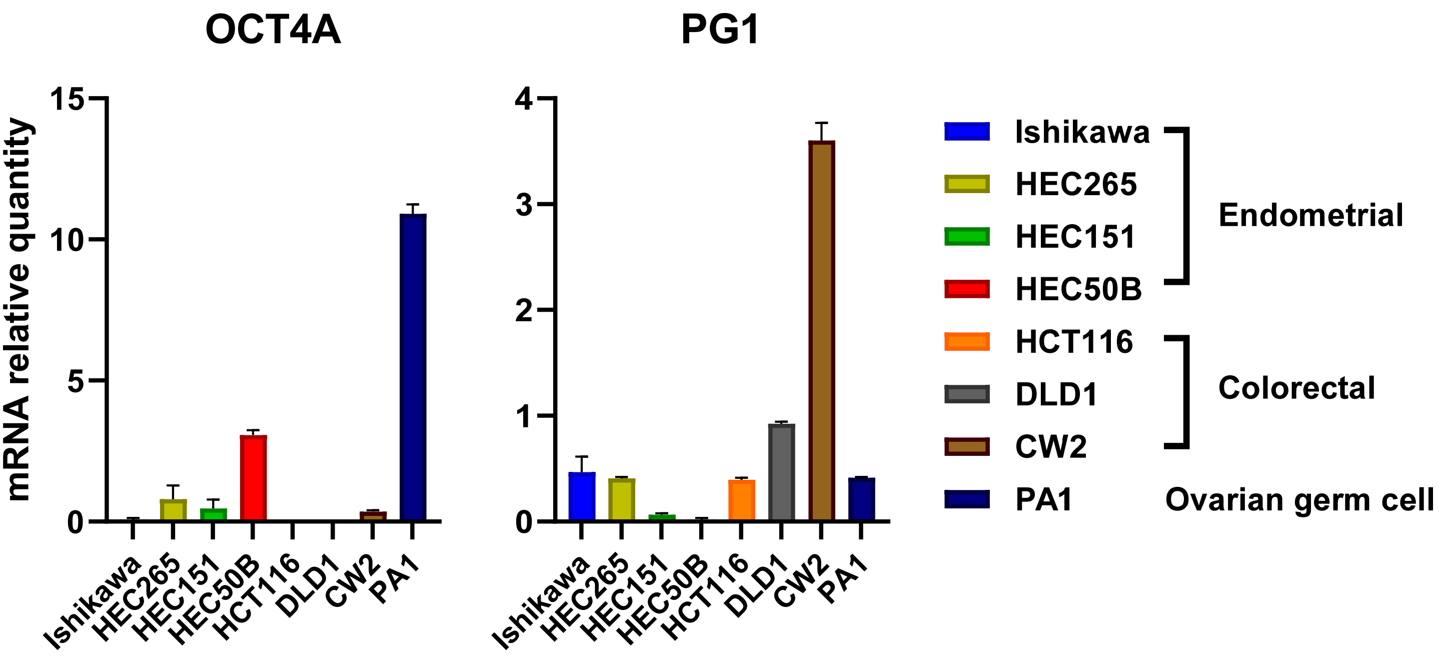

Figure S2. Gene expression of OCT4A and PG1 in endometrial, colorectal and ovarian germ cell tumor cell lines.

Relative mRNA levels of OCT4A and PG1 were measured by real-time PCR using specific primer sets. Data are presented as relative expression levels normalized to HPRT1, with HPRT1 set as 1. n=3. Mean±SD.

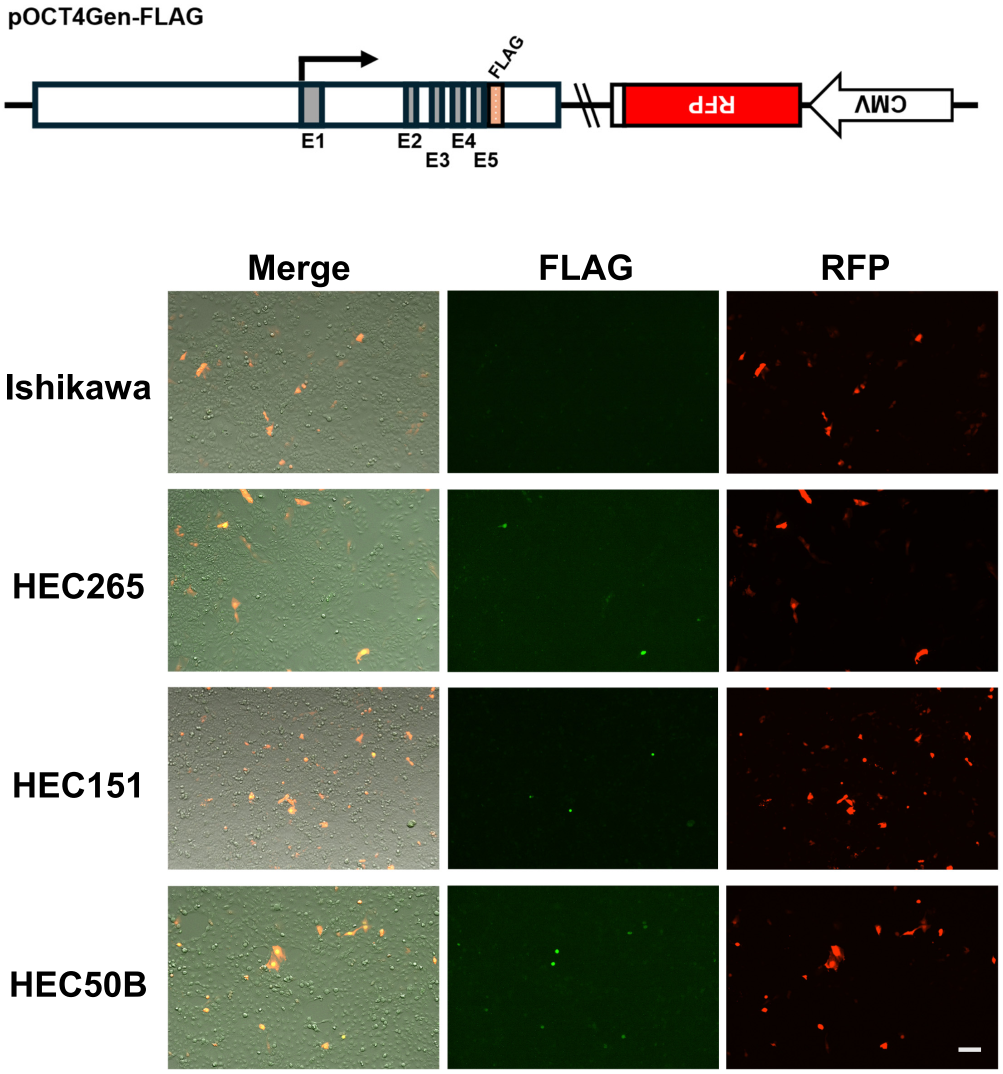

Figure S3. Validation of OCT4A-positive cells in endometrial cancer cell lines using a tagged genomic transgene, related to Figure 1F.

Immunocytochemistry was performed using an anti-FLAG antibody on cells transfected with pOCT4AGen-FLAG. The anti-FLAG antibodies and RFP markers identify FLAG-tagged OCT4A protein-positive cells and vector-transfected cells, respectively. The merged images show the combination of GC-based bright-field, FLAG, and RFP signals. GC refers to gradient contrast images. In all cell lines, the signal of the FLAG-tagged OCT4A protein was observed to localize in the nucleus. Scale bar: 100 µm.

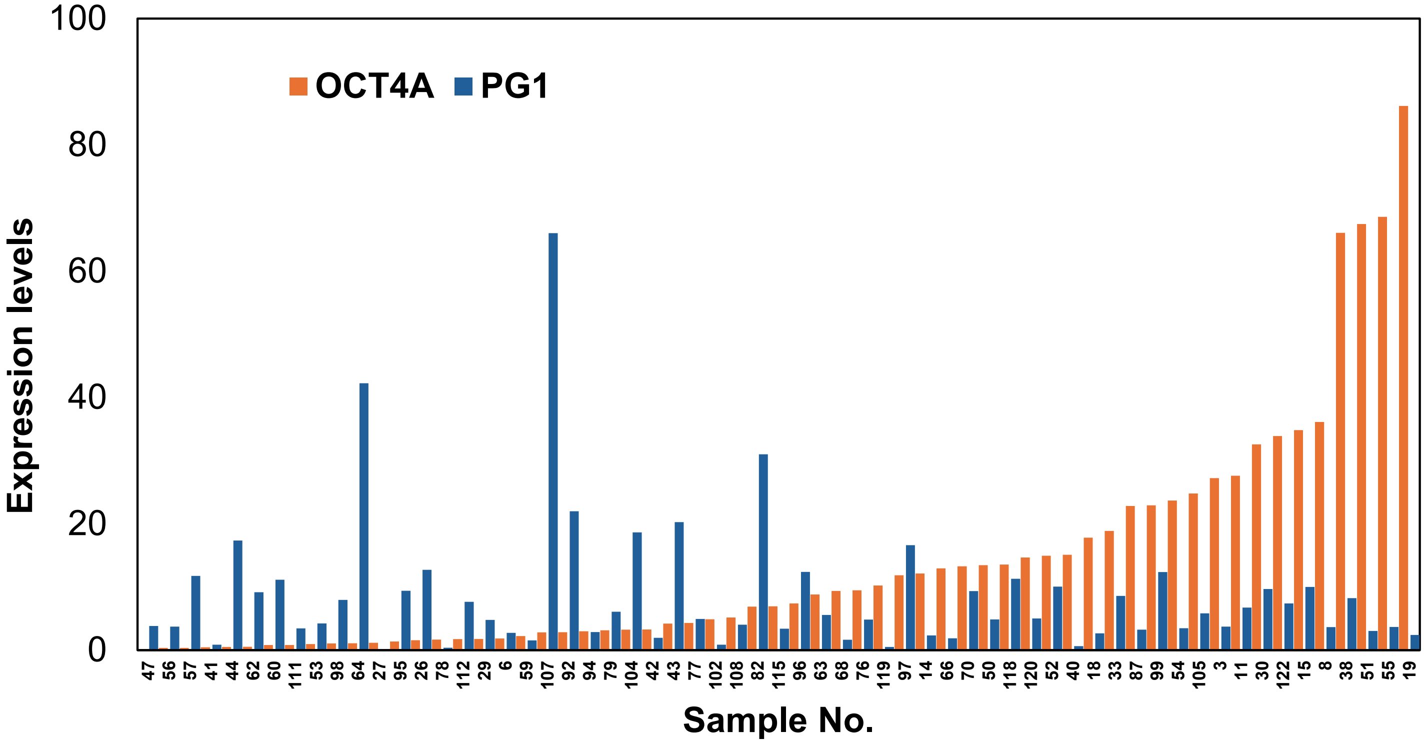

Figure S4. The gene expression levels of OCT4A and PG1 in endometrial cancer tissues by quantitative real–time PCR (qRT–PCR), related to Figure 2.

Expression levels are represented as $2^{-\Delta CT}$ values using HPRT1 as the endogenous control. The graph shows the average values obtained from triplicate well measurements.

A

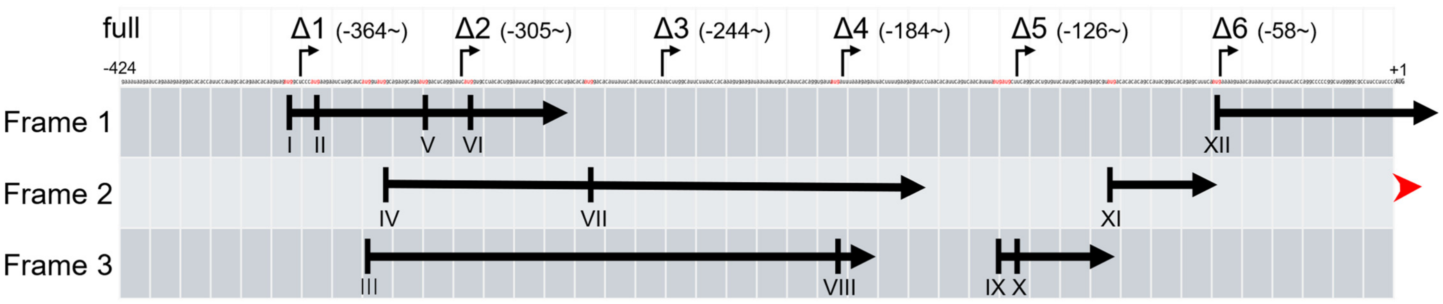

B

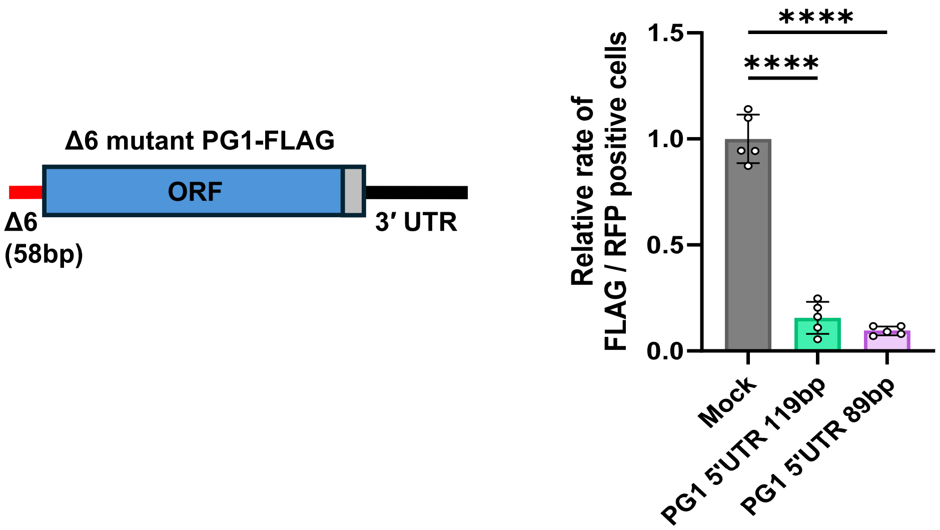

Figure S5. Effect of differences in the length of the PG1 5' UTR sequence on its own translational suppression, related to Figures 3 and 4.

A) The nucleotide sequence of the PG1 5‘ UTR and the potential upstream open reading frame (uORF) are shown. The upper panel presents the lengths and sequences of plasmids used for analysis (full and the six mutants (Δ 1–Δ 6)). Twelve AUG codons (I–XII) are highlighted in red and the PG1 coding sequence (CDS) start codon is shown at +1 position. The lower panel figure illustrates the predicted translation product from the uORF within the 5' UTR, with an arrow pointing to the stop codon. The vertical lines indicate the position of AUG codons (I–XII). Additionally, the start codon of the CDS is indicated by a red arrowhead in frame 2. Translation repression was not observed in the -58 to -1 UTR due to the absence of an AUG codon (Δ 6). In contrast, the -184 to -1 UTR contains a uORF start codon (XII), and its translation product is expected to overlap with the CDS start codon, thereby inhibiting CDS translation (Δ 4, Δ 5). Unexpectedly, in the -244 to -1 region (Δ 3), which includes a further upstream sequence, translation repression was significantly alleviated. The reason for this remains unclear, but it is possible that the presence of start codon VIII affects translation from XII or that it induces an RNA structural change that facilitates ribosome access to the CDS start codon. In the -305 to -1 region (Δ 2), which includes further uORF AUGs (VI, VII), the translation from the AUG at position VIII is suppressed, leading to a level of CDS translation inhibition comparable to that observed in Δ 4 and Δ 5. In the sequence including further upstream regions, the inhibitory effect on CDS translation was pronounced (Δ 1). Notably, in the full-length sequence, PG1 protein translation was almost completely suppressed. The strongest inhibitory effect was observed when the most upstream sequence of the 5' UTR (-424 to -305) was included.

B) The fluorescence intensity of FLAG antibody staining when the Δ 6 (58bp)-PG1-FLAG gene (illustrated) and either the 5′ proximal 119 bp (-424 to -306) or 89 bp (-424 to -336) of the PG1‘s 5′ UTR are simultaneously introduced into NIH3T3 cells. Transfection efficiency was normalized by dividing the fluorescence intensity of the co-transfected pRFP. n=5. Mean ± SD. One-way ANOVA. * = $p < 0.05$; ** = $p < 0.01$; *** = $p < 0.001$; **** = $p < 0.0001$.

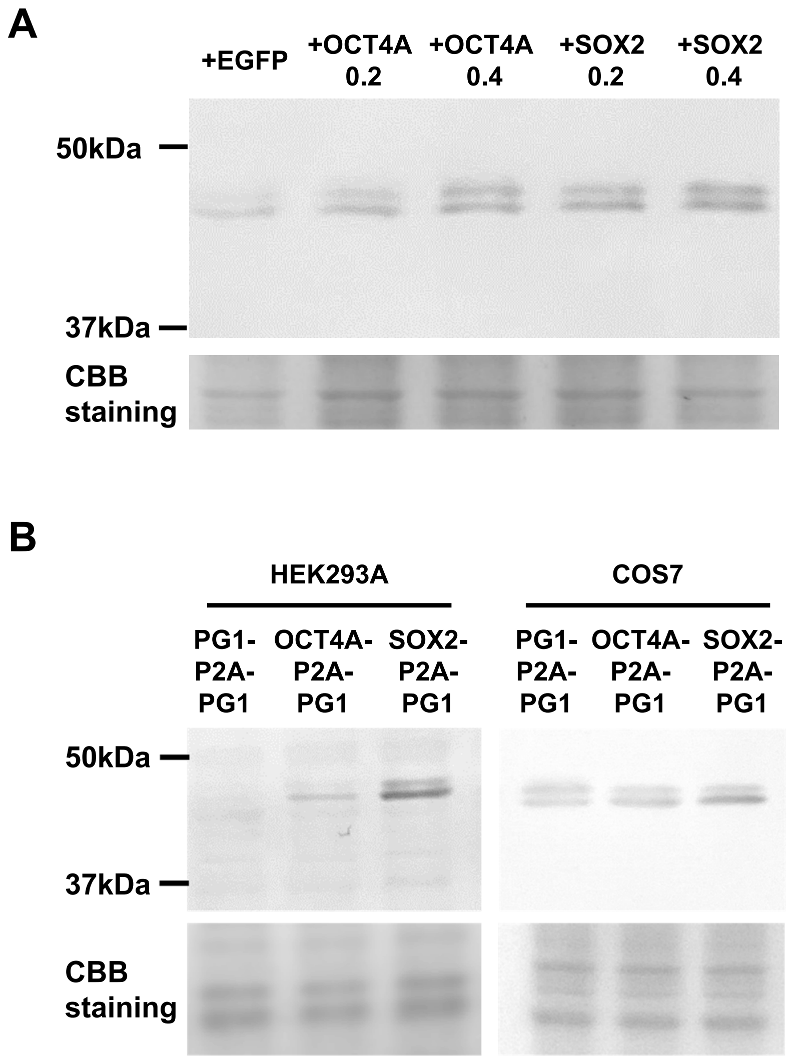

Figure S6. Stabilization of PG1 protein in the presence of stem cell factor OCT4A or SOX2, related to Figure 6.

(A) Expression level of PG1 protein co-transfected with OCT4A or SOX2 in COS7 cells.

(B) Expression levels of PG1 protein co-expressed with OCT4A or SOX2 via a P2A peptide in HEK293A or COS7 cells.

In Western blotting, the acrylamide gel stained with Coomassie Brilliant Blue (CBB) is shown under the panel as a loading control. Uncropped images of Western blot membranes and CBB-stained gels are presented in Figure S9.

```
OCT4A   1   MAGHLASDFAFSPPPGGGGDGPGGPEPGWVDPRTWLSFQGPPGGPGIGPGVGPGSEVWGI   60
            ********************** *.******* ***************************
PG1     1   MAGHLASDFAFSPPPGGGGDGPWGAEPGWVDPLTWLSFQGPPGGPGIGPGVGPGSEVWGI   60

       61   PPCPPPYEFCGGMAYCGPQVGVGLVPQGGLETSQPEGEAGVGVESNSDGASPEPCTVTPG  120
            ********.***************************.**********.*********.**
       61   PPCPPPYELCGGMAYCGPQVGVGLVPQGGLETSQPESEAGVGVESNSNGASPEPCTVPPG  120

      121   AVKLEKEKLEQNPEESQDIKALQKELEQFAKLLKQKRITLGYTQADVGLTLGVLFGKVFS  180
            **************.**********************************.**********
      121   AVKLEKEKLEQNPEKSQDIKALQKELEQFAKLLKQKRITLGYTQADVGLILGVLFGKVFS  180

      181   QTTICRFEALQLSFKNMCKLRPLLQKWVEEADNNENLQEICKAETLVQARKRKRTSIENR  240
            **********************************************.*************
      181   QTTICRFEALQLSFKNMCKLRPLLQKWVEEADNNENLQEICKAETLMQARKRKRTSIENR  240

      241   VRGNLENLFLQCPKPTLQQISHIAQQLGLEKDVVRVWFCNRRQKGKRSSSDYAQREDFEA  300
            ****************** *****************************************
      241   VRGNLENLFLQCPKPTLQ-ISHIAQQLGLEKDVVRVWFCNRRQKGKRSSSDYAQREDFEA  299

      301   AGSPFSGGPVSFPLAPGPHFGTPGYGSPHFTALYSSVPFPEGEAFPPVSVTTLGSPMHSN  360
            ************* *****************************.******.*********
      300   AGSPFSGGPVSFPPAPGPHFGTPGYGSPHFTALYSSVPFPEGEVFPPVSVITLGSPMHSN  359
```

Figure S7. Amino acid compatibility of OCT4A and PG1 proteins, related to Figure 7.

The amino acid sequence of OCT4A and the different amino acids in PG1 are illustrated. OCT4A consists of 360 amino acid residues, while PG1 consists of 359 amino acid residues. An asterisk (*) indicates identical amino acids, and a dot (.) indicates amino acids with high similarity. Blue and red letters represent amino acids that differ between OCT4A and PG1. The yellow highlight indicates the absence of Q259. The amino acid identity between the two is 96%.

(Figure 4E)

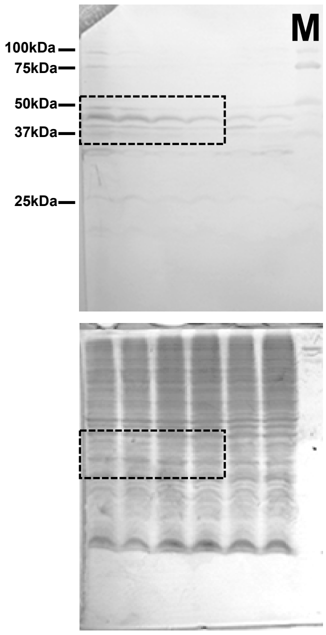

(Figure 5E)

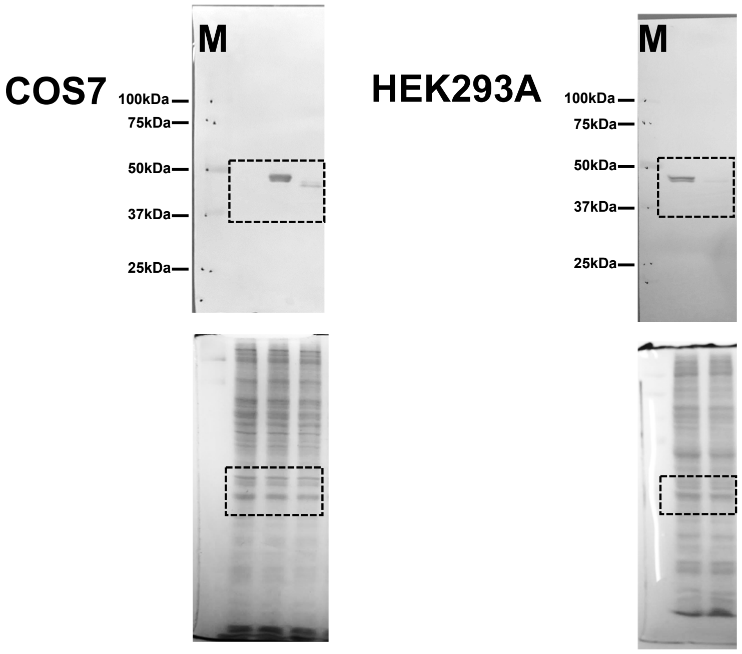

(Figure 5H)

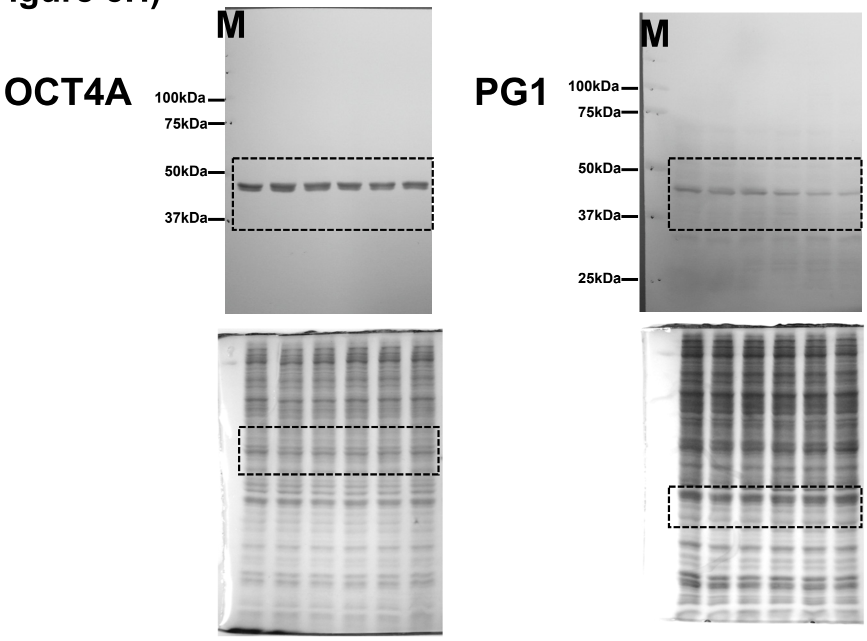

(Figure 5I)

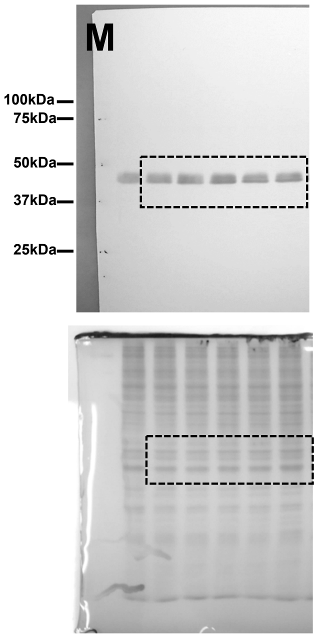

Figure S8. Uncropped images of western blot membranes and CBB-stained gels, related to Figures 4 and 5.

Top: Representative membrane image visualized with BCIP/NBT substrate. Bottom: Coomassie Brilliant Blue (CBB)-stained gel post-transfer, used as a loading control to confirm that equivalent amounts of total protein were loaded into each lane. Black dashed boxes indicate the specific areas cropped for the final figures presented in the main text. Molecular weight marker lanes are labeled as "M" with specific molecular weights (kDa) indicated on the left.

(Figure 7B)

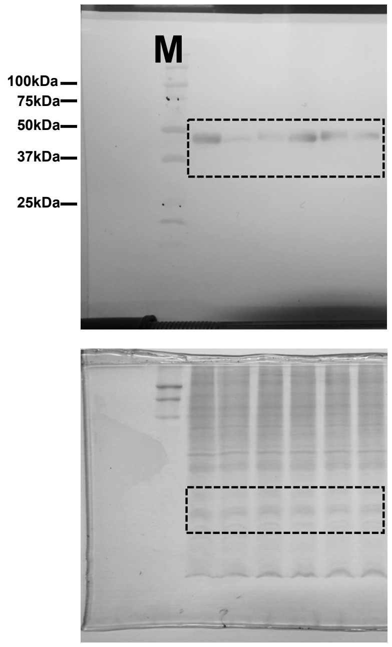

(Figure 7E)

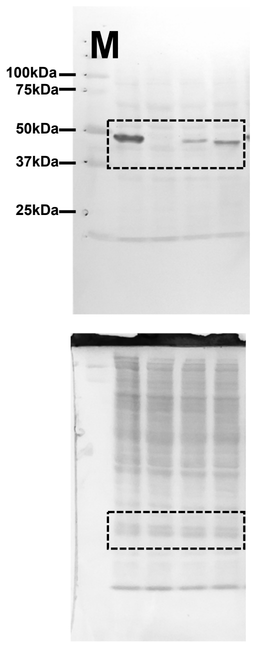

(Figure S6)

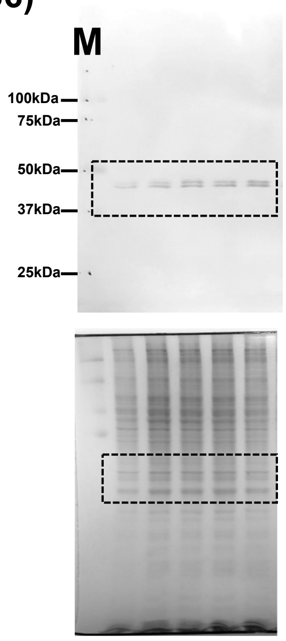

Figure S9. Uncropped images of western blot membranes and CBB-stained gels, related to Figures 7 and S6.
Top: Representative membrane image visualized with BCIP/NBT substrate. Bottom: Coomassie Brilliant Blue (CBB)-stained gel post-transfer, used as a loading control to confirm that equivalent amounts of total protein were loaded into each lane. Black dashed boxes indicate the specific areas cropped for the final figures presented in the main text. Molecular weight marker lanes are labeled as "M" with specific molecular weights (kDa) indicated on the left.

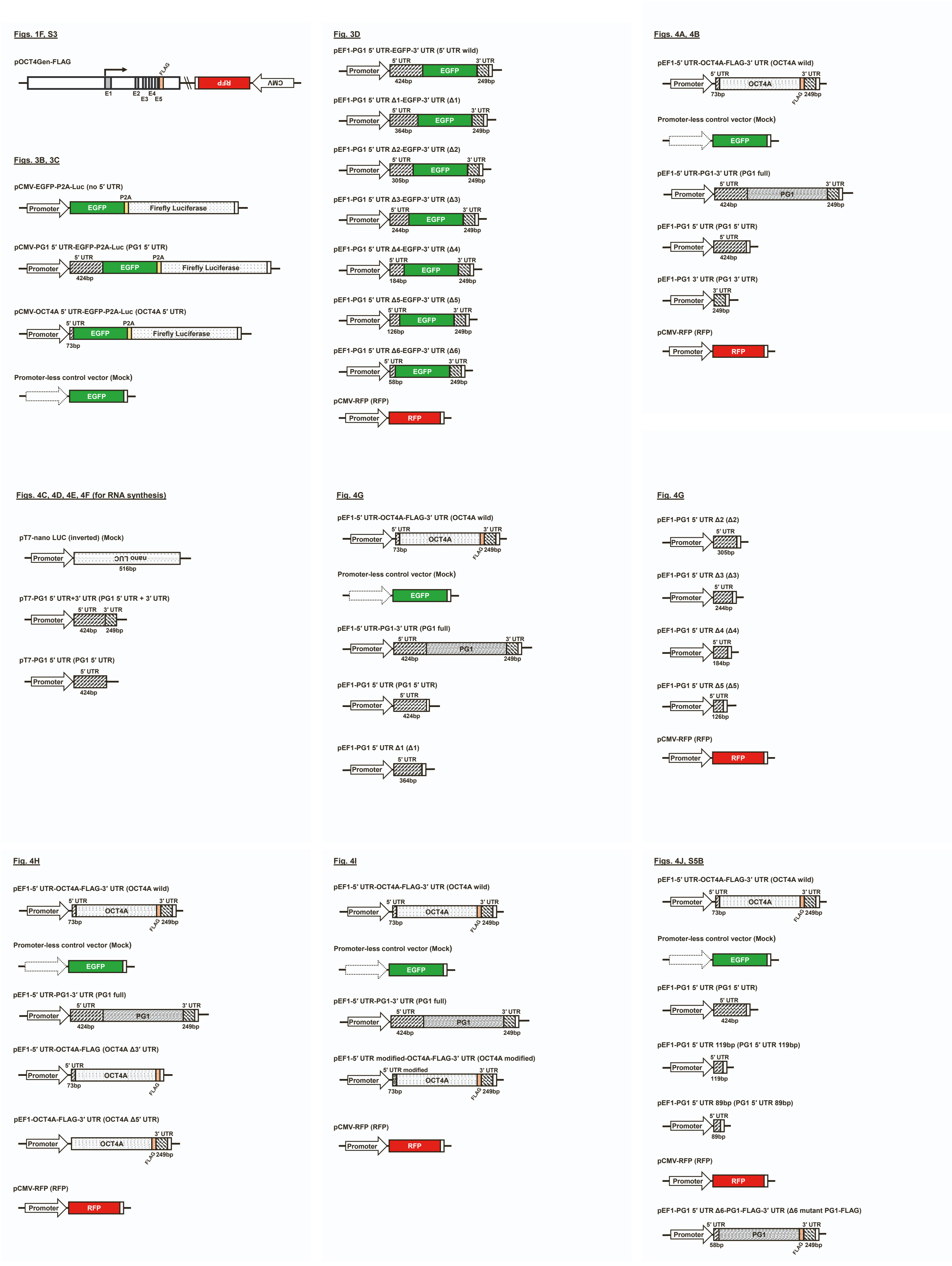

Figure S10. DNA constructs used in this study, related to Figures 1, 3, 4, S3 and S5.

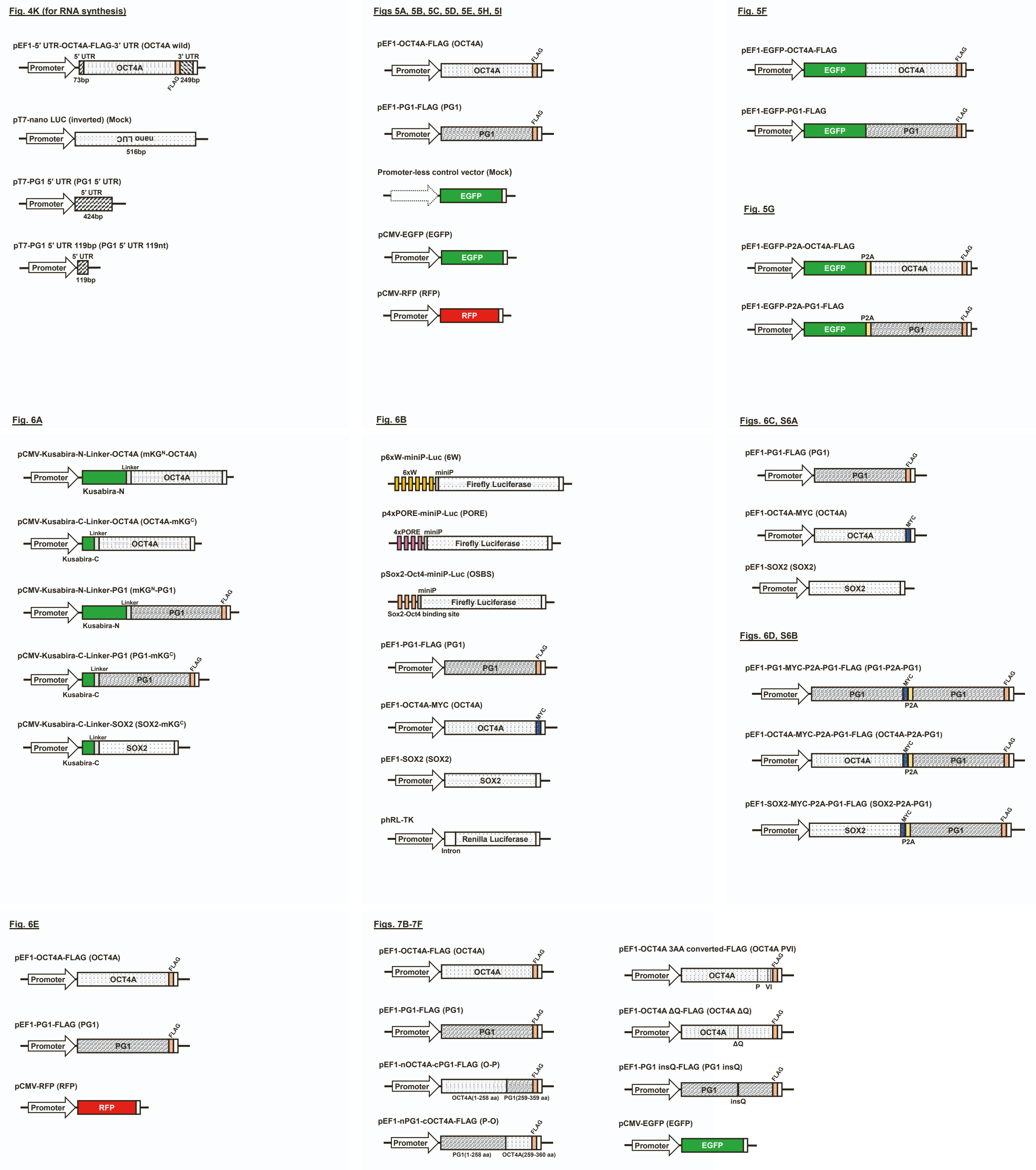

Figure S11. DNA constructs used in this study, related to Figures 4, 5, 6, 7 and S6.

		(N=61)
Age	Median (range)-yr	57 (36-84）
Obesity	Present	28
	Absent	33
DM	Present	9
	Absent	52
Parity	0	10
	1	14
	2	25
	3	12
Menopause	Present	40
	Absent	21
Hormone therapy for breast cancer	Present	5
	Absent	56

Histological subtype	Endometrioid	40
	Endometrioid with SQ	21
FIGO Staging	IA	44
	IB	17
FIGO Tumor grading	Grade 1	45
	Grade 2	7
	Grade 3	9
MI	≧1/2	17
	<1/2	24
	None	20
LVSI	Present	5
	Absent	56
MELF	Present	8
	Absent	53
Malignant ascites	Present	5
	Absent	56

Table S1. Patient background information and pathological findings in a clinical sample of 61 patients, related to Figure 2.

In the FIGO staging system, stage IA indicates that the tumor is limited to the endometrium or invades less than half of the myometrium. Stage IB signifies that the tumor invades one half or more of the myometrium. In terms of tumor differentiation grade: grade1 (G1) tumors exhibit 5% or less solid non-glandular, non-squamous growth. Grade2 (G2) tumors exhibit between 6% and 50% solid non-glandular, non-squamous growth. Grade3 (G3) tumors exhibit more than 50% solid non-glandular, non-squamous growth. The presence of severe cytological atypia in the majority of cells (more than 50%) increases the grade by one level.
DM=diabetes mellitus, SQ=squamous differentiation, FIGO=International Federation of Gynecology and Obstetrics, LVSI=Lymphovascular space invasion, MI=Myometrial invasion, MELF=Microcystic, elongated, and fragmented.
The MELF pattern of invasion shows strong invasion in the tumor tissue and a high frequency of vascular invasion and lymph node metastases (micro metastases), but the impact on long-term prognosis is not clear.

Entry	Reviewed	Organism	Length	Sequence
P20263	reviewed	Mus musculus (Mouse)	352	MAGHLASDFAFSPPPGGGDGSAGLEPGWVDPRTWLSFQGPPGGPGIGPGSEVLGISPCPPAYEFCGGMAYCGPQVGLGLVPQVGVETLQPEGQAGARVESNSEGTSSEPCADRPNAVKLEKVEPTPEESQDMKALQKELEQFAKLLKQKRITLGYTQADVGLTLGVLFGKVFSQTTICRFEALQLSLKNMCKLRPLLEKWVEEADNNENLQEICKSETLVQA**RKRKRTSIENRVRWSLETMFLKCPKPSLQQITHIANQLGLEKDVVRVWFCNRRQKGKRSS**IEYSQREEYEATGTPFPGGAVSFPLPPGPHFGTPGYGSPHFTTLYSVPFPEGEAFPSVPVTALGSPMHSN
B0BMU5	unreviewed	Rattus norvegicus (Rat)	352	MAGHLASDFAFSPPPGGGDGSAGLEPGWVDPRTWLSFQGPPSGPGIGPGSEVLGISPCPPAYEFCGGMAYCGPQVGLGLVPQVGVETLQPEGQAGARVESNSEGASSGPCTARPSAVKLEKVEPSPEESQDMKALQKELEQFAKLLKQKRITLGYTQADVGLTLGVLFGKVFSQTTICRFEALQLSLKNMCKLRPLLEKWVEEADNNENLQEICKSETLVQA**RKRKRTSIENRVRWNLENMFLQCPKPSLQQITSIAKQLGLERDVVRVWFCNRRQKGKRSS**IEYSQREEYEAAGKPFPGGAVSFPLPPGPHFGAPGYGSPHFTTLYSVPFPEGEAFPSVPVTALGSPMHSN
A2ICN2	unreviewed	Oryctolagus cuniculus (Rabbit)	360	MAGHLASDFAFSPPPGGGGDGPGGPEPGWVDPRTWLSFQGPPGGPAIGPGVAPGAEVWGIPPCPPPYDFCGAMAHCAPQLAVGLVPQGGLETSQPEGEAGAGAGSLSEGPSPEPCAAPLGAVKLEKEKLEQTPEESQDMKALQKELEQFAKLLKQKRITLGYTQADVGLTLGVLFGKVFSQTTICRFEALQLSFKNMCKLRPLLQKWVEEADNNENLQEICKAETLVQA**RKRKRTSIENRVRGNLENMFLQCPKPTLQQISHIAQQLGLEKDVVRVWFCNRRQKGKRSS**SDCSQREDFEATGSPFAGGPMSFPLAPGPHFGTPGYGSPHFATLYSPMPFPEGEAFPSVPVPALGSPMHSN
W5PJV9	unreviewed	Ovis aries (Sheep)	360	MAGHLASDFAFSPPPGGGGDGPGGPEPGWVDPRTWMSFQGPPGGSGIGPGVVPGAEVWGLPPCPPPYDLCGGMAYCAPQVGVGPVPPGGLETPQPEGEAGAGVESNSEGASPDPCAAPAGAAKLDKEKLEPNPEESQDIKALQKDLEQFAKLLKQKRITLGYTQADVGLTLGVLFGKVFSQTTICRFEALQLSFKNMCKLRPLLQKWVEEADNNENLQEICKAETLVQA**RKRKRTSIENRVRGNLESMFLQCPKPTLQQISHIAQQLGLEKDVVRVWFCNRRQKGKRSS**SDYSQREDFEAAGSPFAGGPVSFPLAPGPPFGTPGYGGPHFTTLYSSVPFPEGEAFPSVSVTALGSPMHSN
O97552	reviewed	Bos taurus (Bovine)	360	MAGHLASDFAFSPPPGGGGDGPGGPEPGWVDPRTWMSFQGPPGGSGIGPGVVPGAEVWGLPPCPPPYDLCGGMAYCAPQVGVGPVPPGGLETPQPEGEAGAGVESNSEGASPDPCAAPAGAPKLDKEKLEPNPEESQDIKALQKDLEQFAKLLKQKRITLGYTQADVGLTLGVLFGKVFSQTTICRFEALQLSFKNMCKLRPLLQKWVEEADNNENLQEICKAETLVQA**RKRKRTSIENRVRGNLESMFLQCPKPTLQQISHIAQQLGLEKDVVRVWFCNRRQKGKRSS**SDYSQREDFEAAGSPFTGGPVSSPLAPGPHFGTPGYGGPHFTTLYSSVPFPEGEVFPSVSVTALGSPMHAN
Q9TSV5	reviewed	Sus scrofa (Pig)	360	MAGHLASDFAFSPPPGGGGDGPGGPEPGWVDPRTWLSFQGPPGGSGIGPGVGPGAEVWGLPACPPPYDFCGGMAYCAPQVGVGLVPQGGLETPQPEGEAGAGVESNSEGASPEPCAAPAGAAKLDKEKLEPNPEESQDIKALQKDLEQFAKLLKQKRITLGYTQADVGLTLGVLFGKVFSQTTICRFEALQLSFKNMCKLRPLLQKWVEEADNNENLQEICKAETLVQA**RKRKRTSIENRVRGNLESMFLQCPKPTLQQISHIAQQLGLEKDVVRVWFCNRRQKGKRSS**SDYSQREDFEAAGSPFPGGPVSFPLAPGPHFGTPGYGGPHFTTLYSSVPFPEGEAFPSVSVTPLGSPMHSN
A0A6P5J5Z9	unreviewed	Phascolarctos cinereus (Koala)	344	MAGHLAPEYFSPPPGGGGAGGSEPTWNWPGFQGPPGGAGTGPGPDGWGMAPYHPLYDMWGGGVYCEPQPNVGVMAQPAQEIAVPDGDAGPGVESPSEGSSPEPRATGRVTKIEPVESGDEQPQETPSPEELEQFAKELKRKRITLGYTQADVGITLGALFGKVFSQTTICRFEALQLSFKNMCKLRPLLQKWLEAADDNDHLQELCKAETVLQQA**RKRKRTSIENGVRGNLETMFLQCPKPTLQQISNIAEELGLEKDVVRVWFCNRRQKGKRSN**SNSPREDFEAAGSFPGAPMPFPLAPGPPFSHPSYGGPHFTTLYSPAPFTEGDAFSSLPVTPLGSTMHSS
A0A8C0MBD5	unreviewed	Canis lupus familiaris (Dog) (Canis familiaris)	360	MAGHLASDLAFSPSPGGGGDGPGGPDPGWGDPRAWLSFPGPPGGPALGPGVGPGAEVWGLPPCPPPYEFCGGMAYCGPQVGVGLLPQGGLDTSQPEGERGAGLEGSSEGASPEPCAAPPGVVKPDKEKLEQNPEESQDIKALQKDLEQFAKLLKQKRITLGYTQADVGLTLGVLFGKVFSQTTICRFEALQLSFKNMCKLRPLLQKWVEEADNNENLQEICKAETLVQA**RKRKRTSIENRVRGNLENMFLQCPKPTLQQISHIAQQLGLEKDVVRVWFCNRRQKGKRSS**SDYSQREDFEAAGSPFSGAPVSFPLAPGPHFGTPGYGGPHFTTLYSSVPLPEGEGFPSVSVTTLGSPMHSN
F6Y386	unreviewed	Equus caballus (Horse)	360	MAGHLASDFAFSPPPGGGGDGPGGPEPGWVDPRTWLSFQGPPSGSGIGPGVGPGAEVWGIPPCPPPYEFCGGMAYCGPQVGVGLVPQGSLETSQPEGEAGARVESNSEGASPEPCAAPPGAVKVDKEKLEQNPEESQDIKALQKDLEQFAKLLKQKRITLGYTQADVGLTLGVLFGKVFSQTTICRFEALQLSFKNMCKLRPLLQKWVEEADNNENLQEICKAETLVQA**RKRKRTSIENRVRGNLENMFLQCPKPTLQQISHIAQQLGLEKDVVRVWFCNRRQKGKRSS**SDYSQREDFEAAGSPFSGGPVSFPLAPGPHFGTPGYGGPHFTTLYSSVPFPEGEAFPSVSVTTLGSPMHSN
A0A8C0DFW7	unreviewed	Balaenoptera musculus (Blue whale)	360	MAGHLASDFAFSPPPGGGGDGPGGPEPGWVDPRTWMSFQGPPGGSGIGPGVGPGAEVWGLPACPPPYDFCGGMAYCAPQVGVGLVPQGGLETPQPEGEAGAGVESNSEGASPEPCAAPAGPVKLDKEKLEPNPEESQDIKALQKDLEQFAKLLKQKRITLGYTQADVGLTLGVLFGKVFSQTTICRFEALQLSFKNMCKLRPLLQKWVEEADNNENLQEICKAETLVQA**RKRKRTSIENRVRGNLESMFLQCPKPTLQQISHIAQQLGLEKDVVRVWFCNRRQKGKRSS**SDYSQREDFEAAGSPFSGGPVSFPLAPGPHFGTPGYGGPHFTTLYSSVPFPEGEAFPSVSVTTLGSPMHSN
A0A8B7QI41	unreviewed	Hipposideros armiger (Great Himalayan leaf-nosed bat)	362	MAGHLASDFAFSPPPGGGGDGPGGPEPGWVDPRTWLSFQGPPGGPGIGPGLGPGAEVWGIPSCPPPYEFCAGMAYCGTQVGVGLVPHGGLETPQPEGEAGAGVESSSEGASPEPCSAPPGAVKLDKEKPEQNPEEEASEDMKALQKDLEQFAKLLKQKRITLGYTQADVGLTLGVLFGKVFSQTTICRFEALQLSLKNMCKLRPLLQKWVEEADNNENLQEICKAETLVQA**RKRKRTSIENRVRGNLESMFLQCPKPTLQQISHIAQQLGLEKDVVRVWFCNRRQKGKRSS**SDYSQREDFEASGPHFSGGPVSFPLAAGPHFGTPGYVGPHFTTLYSSVPFPEGDAFPSVSVATLGSPMHSN
Q5TM49	reviewed	Macaca mulatta (Rhesus macaque)	360	MAGHLASDFAFSPPPGGGGDGPGGPETGWVDPRTWLSFQGPPGGPGIGPGVGPGSEVWGIPPCPPPYEFCGGMAYCGPQVGVGLVPQGGLETSQPEGEAGAGVESNSDGASPEPCTVPTGAVKLEKEKLEQNPEESQDIKALQKELEQFAKLLKQKRITLGYTQADVGLTLGVLFGKVFSQTTICRFEALQLSFKNMCKLRPLLQKWVEEADNNENLQEICKAETLVQA**RKRKRTSIENRVRGSLENLFLQCPKPTLQQISHIAQQLGLEKDVVRVWFCNRRQKGKRSS**SDYAQREDFEAAGSPFSGGPVSFPLAPGPHFGTPGYGSPHFTALYSSVPFPEGEAFPPVPVTTLGSPMHSN
A0A6D2YBI4	unreviewed	Pongo abelii (Sumatran orangutan) (Pongo pygmaeus abelii)	360	MAGHLASDFAFSPPPGGGGDGPGGPEPGWVDPRTWLSFQGPPGGPGIGPGVGPGSEVWGIPPCPPPYEFCGGMAYCGPQVGVGLVPQGSLETSQPEGEAGVGVESNSDGASPEPCTVPPGAVKLEKEKLEQNPEESQDIKALQKELEQFAKLLKQKRITLGYTQADVGLTLGVLFGKVFSQTTICRFEALQLSFKNMCKLRPLLQKWVEEADNNENLQEICKAETLVQA**RKRKRTSIENRVRGNLENLFLQCPKPTLQQISHIAQQLGLEKDVVRVWFCNRRQKGKRSS**SDYAQREDFEAAGSPFSGGPVSFPLAPGPHFGTPGYGSPHFTALYSSVPFPEGEAFPPVSVTTLGSPMHSN
G3S2Z8	unreviewed	Gorilla gorilla gorilla (Western lowland gorilla)	360	MAGHLASDFAFSPPPGGGGDGPGGPEPGWVDPRTWLSFQGPPGGPGIGPGVGPGSEVWGIPPCPPPYEFCGGMAYCGPQVGVGLVPQGGLETSQPEGEAGVGVESNSDGASPEPCTVTPGAVKLEKEKLEQNPEESQDIKALQKELEQFAKLLKQKRITLGYTQADVGLTLGVLFGKVFSQTTICRFEALQLSFKNMCKLRPLLQKWVEEADNNENLQEICKAETLVQA**RKRKRTSIENRVRGNLENLFLQCPKPTLQQISHIAQQLGLEKDVVRVWFCNRRQKGKRSS**SDYAQREDFEAAGSPFSGGPVSFPLAPGPHFGTPGYGSPHFTALYSSVPFPEGEAFPPVSVTTLGSPMHSN
Q7YR49	reviewed	Pan troglodytes (Chimpanzee)	360	MAGHLTSDFAFSPPPGGGGDGPGGPEPGWVDPRTWLSFQGPPGGPGIGPGVGPGSEVWGIPPCPPPYEFCGGMAYCGPQVGVGLVPQGGLETSQPEGEAGVGVESNSDGASPEPCTVTPGAVKLEKEKLEQNPEESQDIKALQKELEQFAKLLKQKRITLGYTQADVGLTLGVLFGKVFSQTTICRFEALQLSFKNMCKLRPLLQKWVEEADNNENLQEICKAETLVQA**RKRKRTSIENRVRGNLENLFLQCPKPTLQQISHIAQQLGLEKDVVRVWFCNRRQKGKRSS**SDYAQREDFEAAGSPFSGGPVSFPLAPGPHFGTPGYGSPHFTALYSSVPFPEGEAFPPVSVTTLGSPMHSN
Q01860	reviewed	Homo sapiens (Human)	360	MAGHLASDFAFSPPPGGGGDGPGGPEPGWVDPRTWLSFQGPPGGPGIGPGVGPGSEVWGIPPCPPPYEFCGGMAYCGPQVGVGLVPQGGLETSQPEGEAGVGVESNSDGASPEPCTVTPGAVKLEKEKLEQNPEESQDIKALQKELEQFAKLLKQKRITLGYTQADVGLTLGVLFGKVFSQTTICRFEALQLSFKNMCKLRPLLQKWVEEADNNENLQEICKAETLVQA**RKRKRTSIENRVRGNLENLFLQCPKPTLQQISHIAQQLGLEKDVVRVWFCNRRQKGKRSS**SDYAQREDFEAAGSPFSGGPVSFPLAPGPHFGTPGYGSPHFTALYSSVPFPEGEAFPPVSVTTLGSPMHSN

Table S2. Amino acid sequence differences of *POU5F1* protein in mammals.

Amino acid sequences of mammalian *POU5F1* obtained using UniProtKB/TrEMBL are shown. The Q258-Q259 region is highlighted in red. The DNA-binding Homeobox domain is indicated in bold.
